# Supplementary material for: Dynamic hydrogen-bonding enables high-performance and mechanically robust organic solar cells processed with non-halogenated solvent
Source: Nat Commun. 2025 Jan 17;16:787. doi: 10.1038/s41467-024-55375-8 (PMC11748654; doi:10.1038/s41467-024-55375-8)
Supplement: Supplementary file 1 — Supplementary Information [file 41467_2024_55375_MOESM1_ESM.pdf]

Dynamic hydrogen-bonding enables high-performance and mechanically robust organic solar cells processed with non-halogenated solvent

Haozhe He[1,2], Xiaojun Li[1,2]*, Jingyuan Zhang[1], Zekun Chen[1,2], Yufei Gong[1,2], Hongmei Zhuo[1,2], Xiangxi Wu[1,2], Yuechen Li[1,3], Shijie Wang[4], Zhaozhao Bi[4], Bohao Song[5], Kangkang Zhou[6], Tongling Liang[2,7], Wei Ma[4], Guanghao Lu[5], Long Ye[6], Lei Meng[1,2], Ben Zhang[8], Yaowen Li[8] and Yongfang Li[1,2,8]*

1. CAS Key Laboratory of Organic Solids, Institute of Chemistry, Chinese Academy of Sciences, Beijing 100190, China.
2. School of Chemical Science, University of Chinese Academy of Sciences, Beijing 100049, China.
3. School of Materials Science and Engineering, Shaanxi Normal University, Xi'an 710119, China.
4. State Key Laboratory for Mechanical Behavior of Materials, Xi'an Jiaotong University, Xi'an 710049, China.
5. Frontier Institute of Science and Technology, and State Key Laboratory of Electrical Insulation and Power Equipment, Xi'an Jiaotong University, Xi'an, 710054, China.
6. School of Materials Science and Engineering, Tianjin Key Laboratory of Molecular Optoelectronic Sciences, Tianjin University, Tianjin 300350, China.
7. Center for Physicochemical Analysis and Measurement, Institute of Chemistry, Chinese Academy of Sciences, Beijing 100190, China.
8. Laboratory of Advanced Optoelectronic Materials, College of Chemistry, Chemical Engineering and Materials Science, Soochow University, Suzhou, Jiangsu 215123, China.

e-mail: lixiaojun@iccas.ac.cn; liyf@iccas.ac.cn

Table of Contents

Supplementary Figures 5
Supplementary Fig. 1 5
Supplementary Fig. 2 6
Supplementary Fig. 3 6
Supplementary Fig. 4 7
Supplementary Fig. 5 7
Supplementary Fig. 6 8
Supplementary Fig. 7 8
Supplementary Fig. 8 9
Supplementary Fig. 9 9
Supplementary Fig. 10 10
Supplementary Fig. 11 10
Supplementary Fig. 12 11
Supplementary Fig. 13 11
Supplementary Fig. 14 12
Supplementary Fig. 15 12
Supplementary Fig. 16 13
Supplementary Fig. 17 13
Supplementary Fig. 18 14
Supplementary Fig. 19 14
Supplementary Fig. 20 15
Supplementary Fig. 21 15
Supplementary Fig. 22 16
Supplementary Fig. 23 16
Supplementary Fig. 24 17
Supplementary Fig. 25 17
Supplementary Fig. 26 18
Supplementary Fig. 27 18

Supplementary Fig. 28 18
Supplementary Fig. 29 18
Supplementary Fig. 30 19
Supplementary Fig. 31 19
Supplementary Fig. 32 19
Supplementary Fig. 33 19
Supplementary Fig. 34 20
Supplementary Fig. 35 20
Supplementary Fig. 36 21
Supplementary Fig. 37 22
Supplementary Fig. 38 23
Supplementary Fig. 39 24
Supplementary Fig. 40 25
Supplementary Fig. 41 26
Supplementary Fig. 42 27
Supplementary Fig. 43 28
Supplementary Fig. 44 29
Supplementary Fig. 45 30
Supplementary Fig. 46 31
Supplementary Fig. 47 32
Supplementary Fig. 48 33
Supplementary Fig. 49 34
Supplementary Fig. 50 35
Supplementary Fig. 51 36
Supplementary Fig. 52 37
Supplementary Fig. 53 38
Supplementary Fig. 54 39
Supplementary Fig. 55 40
Supplementary Fig. 56 41
Supplementary Tables 42

Supplementary Table 1 42
Supplementary Table 2 43
Supplementary Table 3 43
Supplementary Table 4 43
Supplementary Table 5 44
Supplementary Table 6 45
Supplementary Table 7 45
Supplementary Methods 46
Synthesis of compound E3-1 46
Synthesis of compound E3-2 46
Synthesis of compound E3-3 46
Synthesis of compound E3-4 47
Synthesis of compound E3-5 47
Synthesis of BTA-E3 48
Synthesis of compound E6-2 48
Synthesis of compound E6-3 48
Synthesis of BTA-E6 49
Synthesis of compound E9-2 49
Synthesis of compound E9-3 50
Synthesis of BTA-E9 50
Supplementary Notes 51
Supplementary Note I 51
Supplementary Note II 51
Supplementary References 53

Supplementary Figures

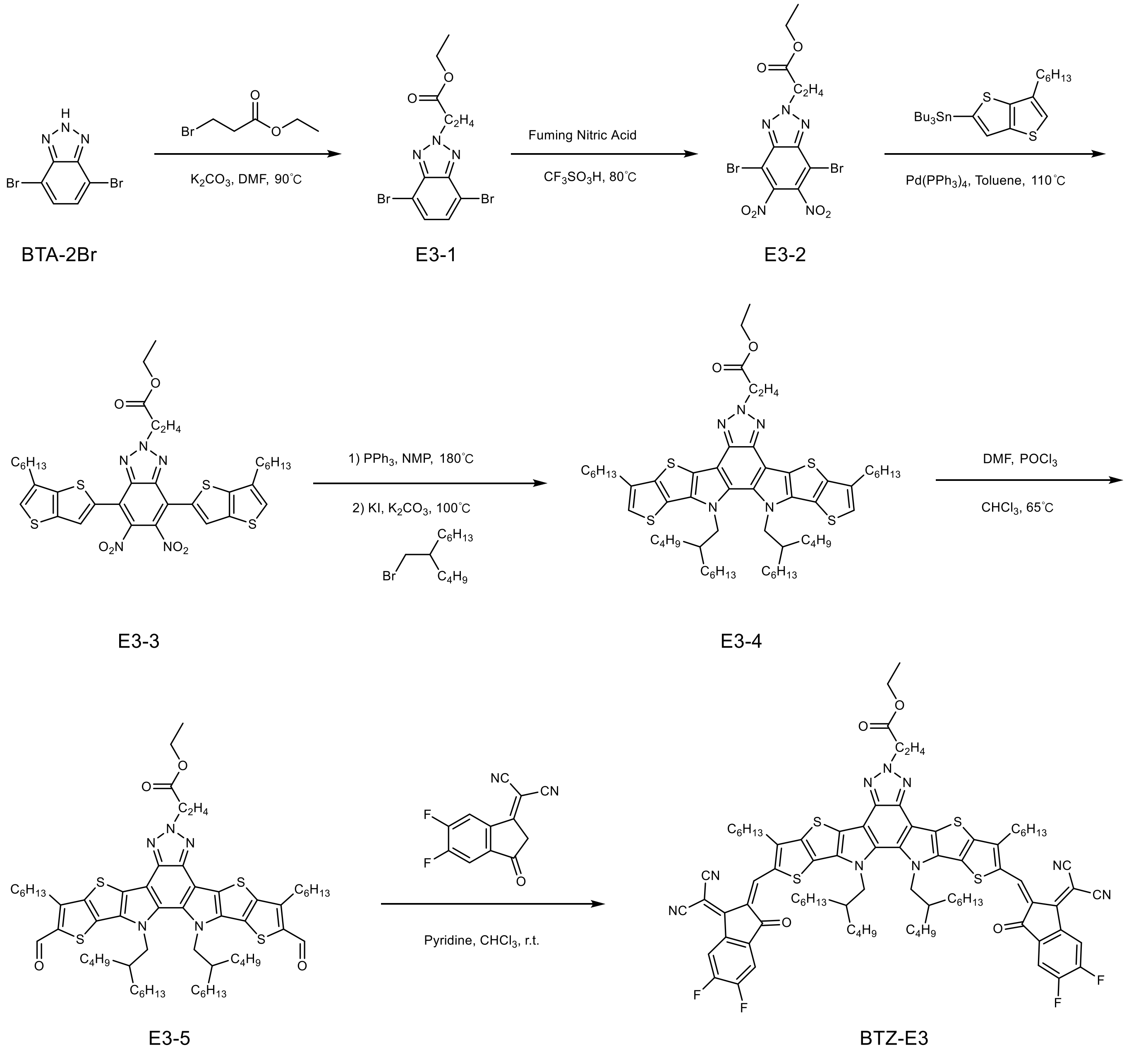

Supplementary Fig. 1 | Synthetic route of BTA-E3.

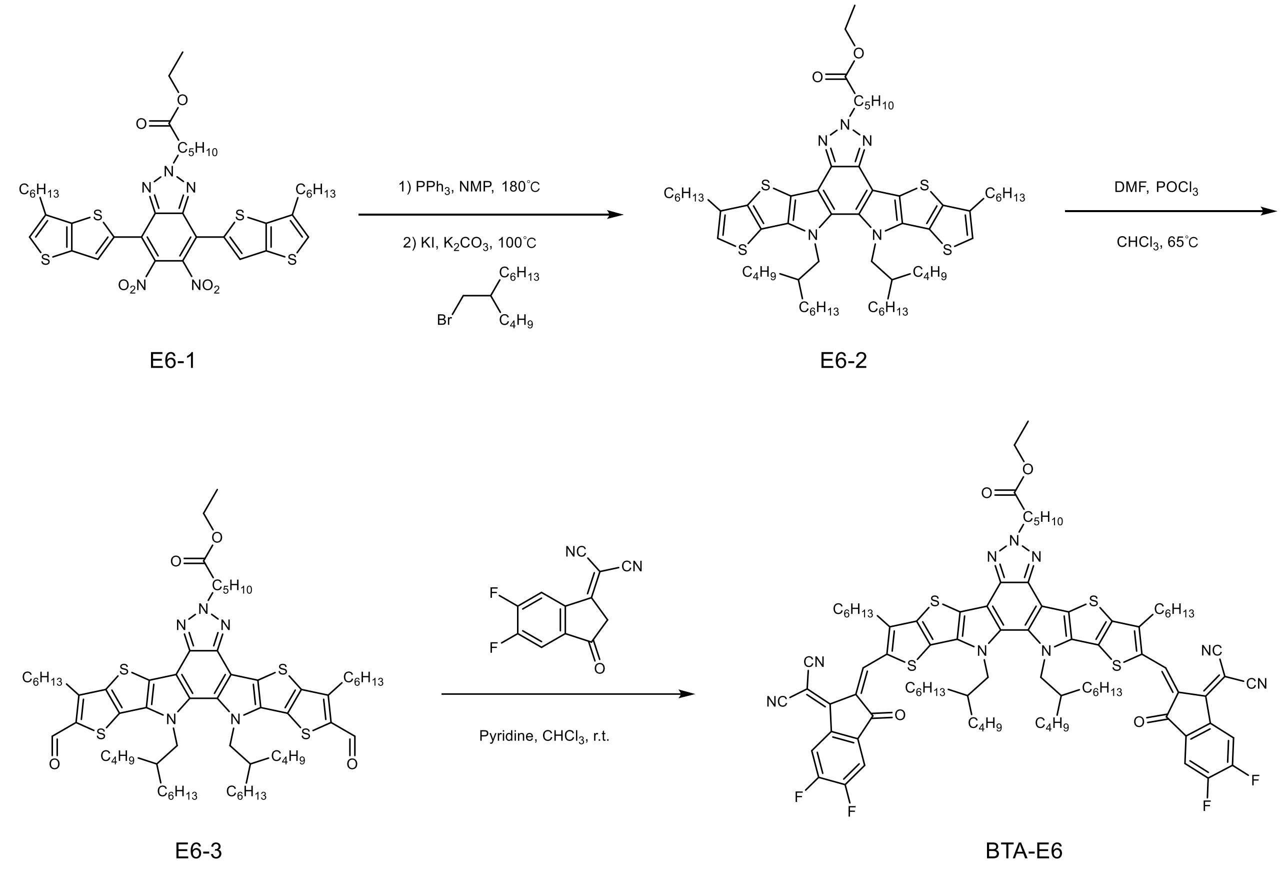

Supplementary Fig. 2 | Synthetic route of BTA-E6.

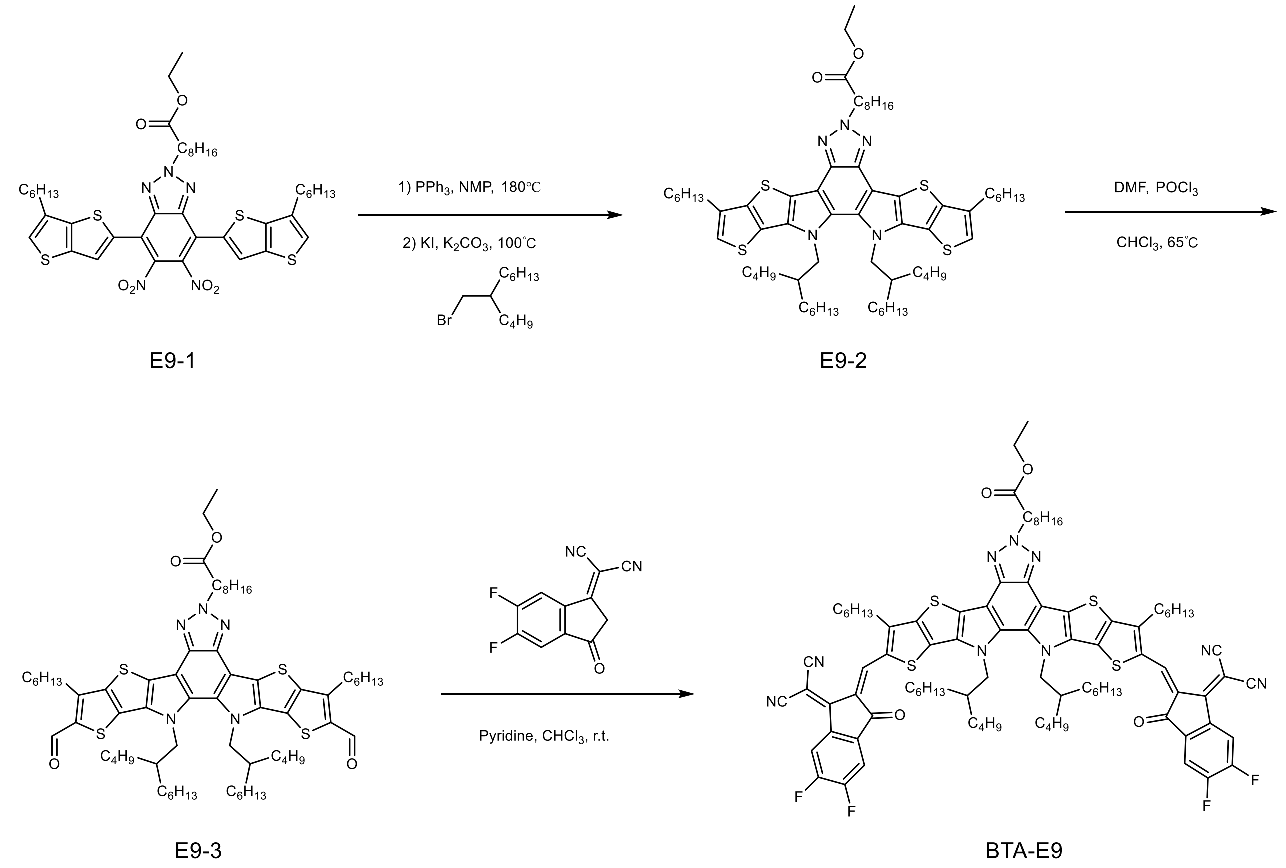

Supplementary Fig. 3 | Synthetic route of BTA-E9.

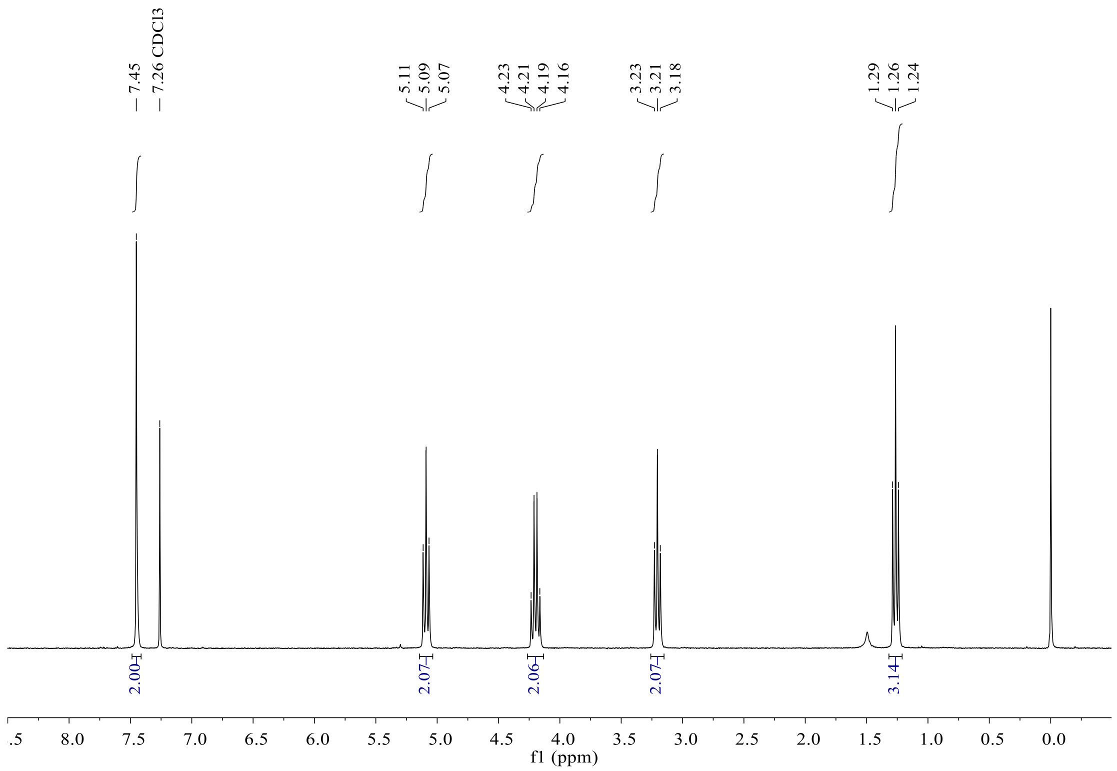

Supplementary Fig. 4 | ^{1}H NMR spectrum of compound E3-1 in $CDCl_3$.

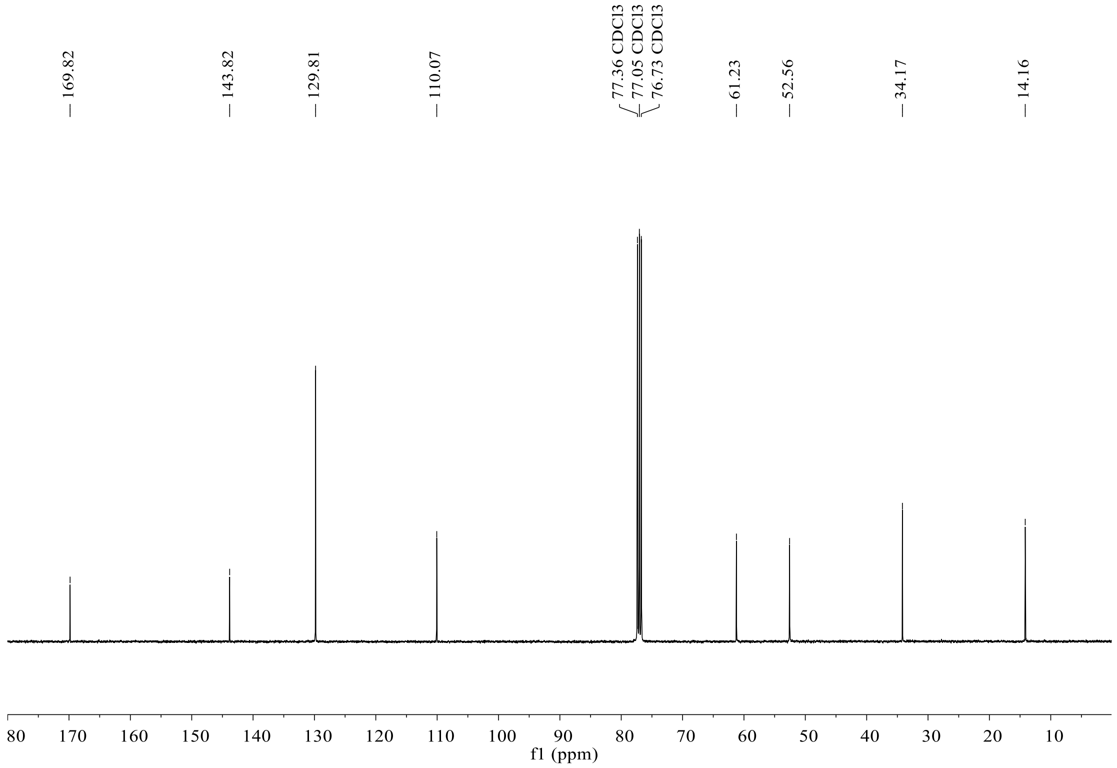

Supplementary Fig. 5 | ^{13}C NMR spectrum of compound E3-1 in $CDCl_3$.

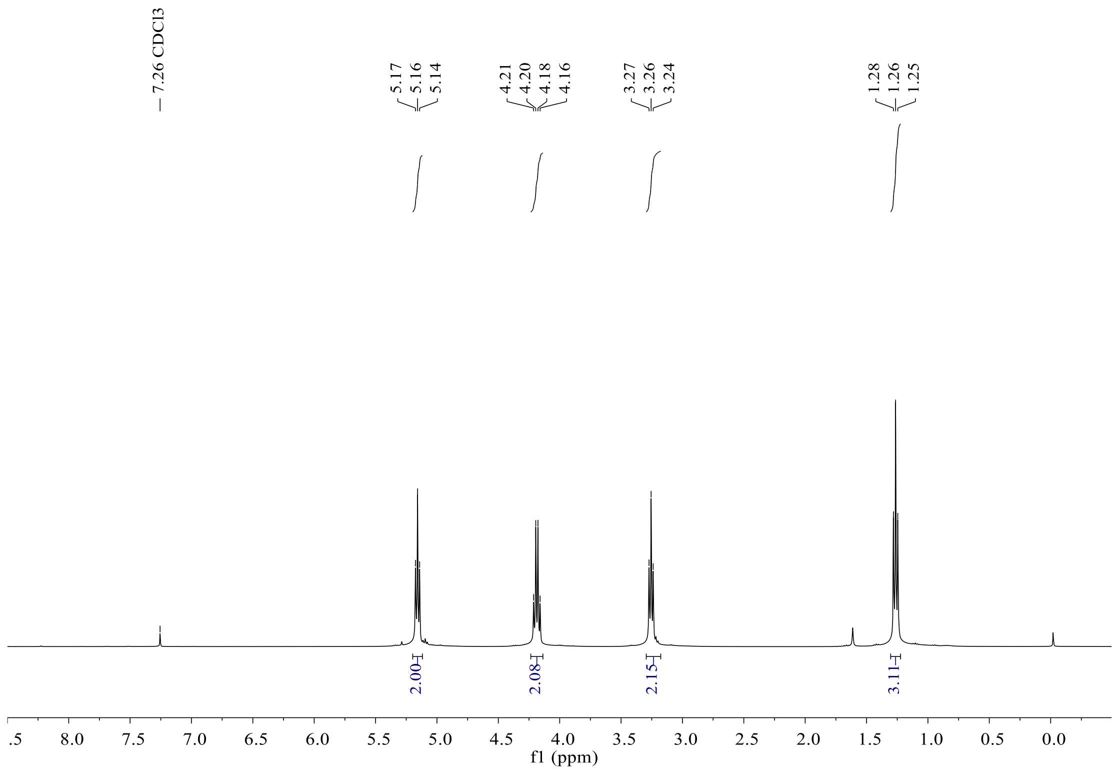

Supplementary Fig. 6 | ^{1}H NMR spectrum of compound E3-2 in $CDCl_3$.

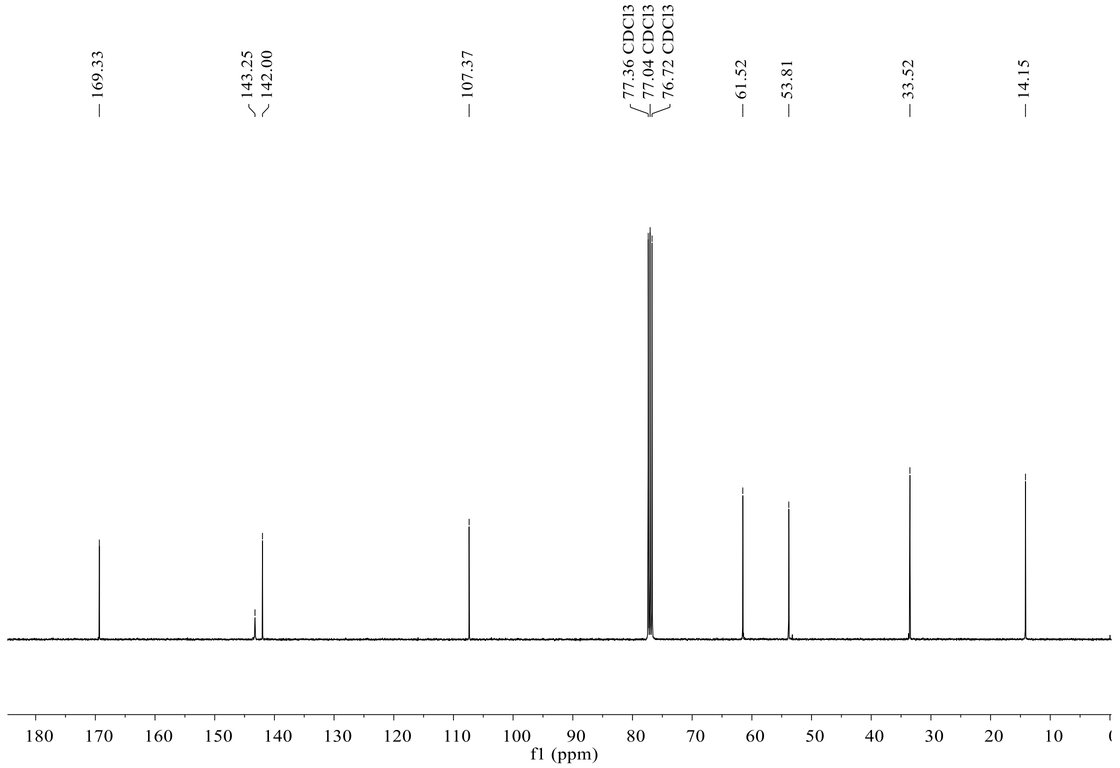

Supplementary Fig. 7 | ^{13}C NMR spectrum of compound E3-2 in $CDCl_3$.

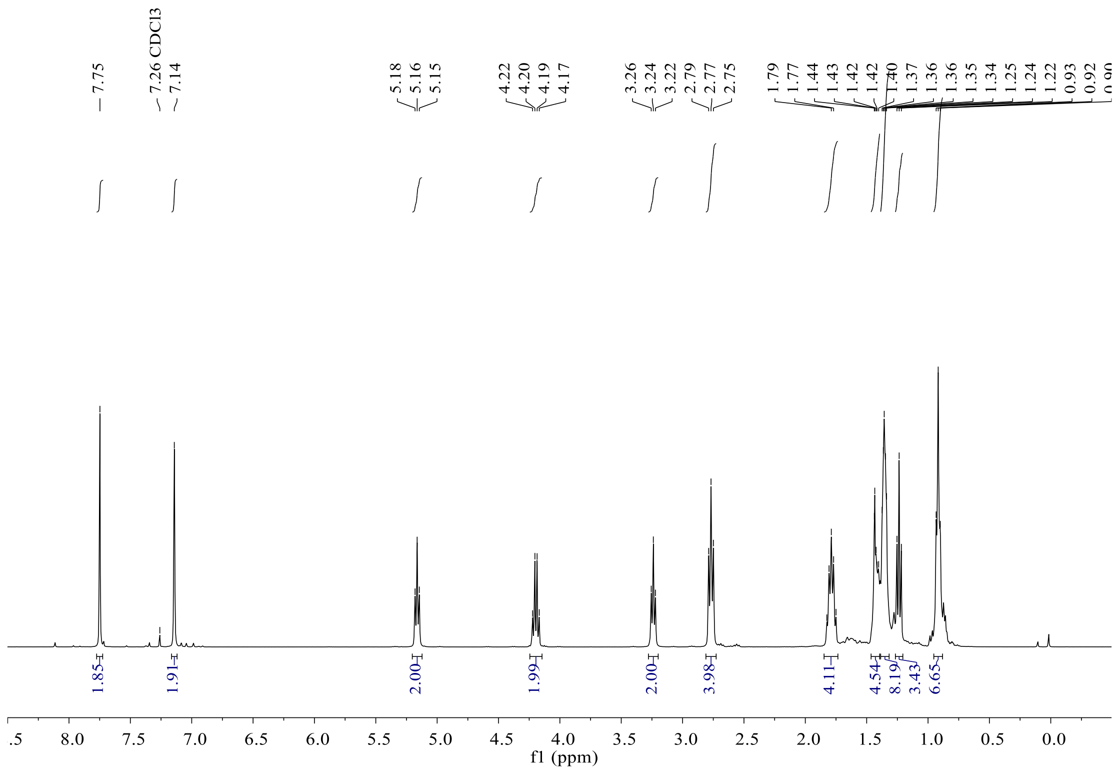

Supplementary Fig. 8 | ^{1}H NMR spectrum of compound E3-3 in $CDCl_3$.

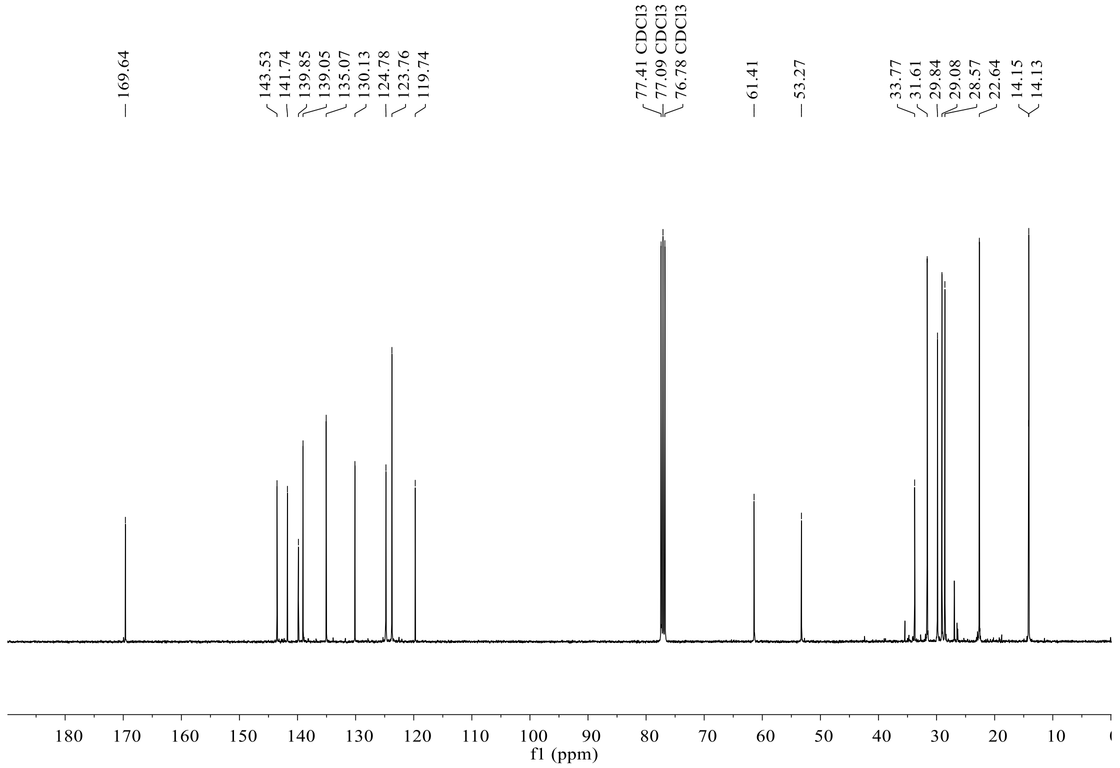

Supplementary Fig. 9 | ^{13}C NMR spectrum of compound E3-3 in $CDCl_3$.

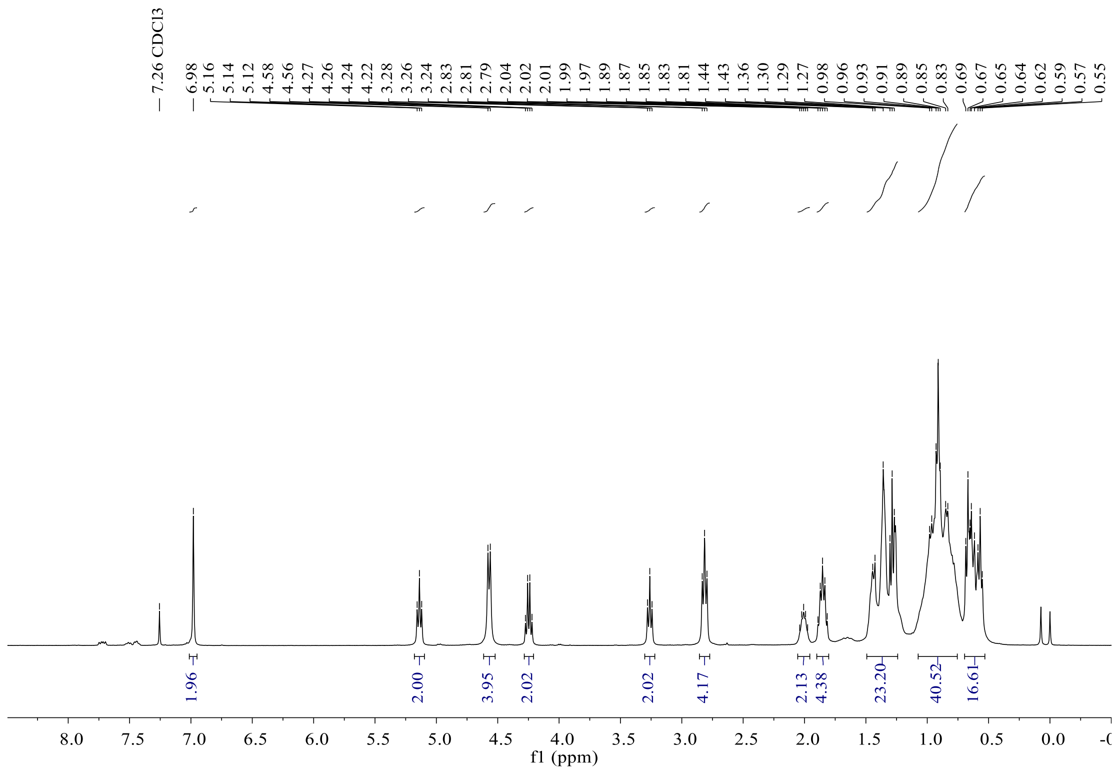

Supplementary Fig. 10 | ^{1}H NMR spectrum of compound E3-4 in $CDCl_3$.

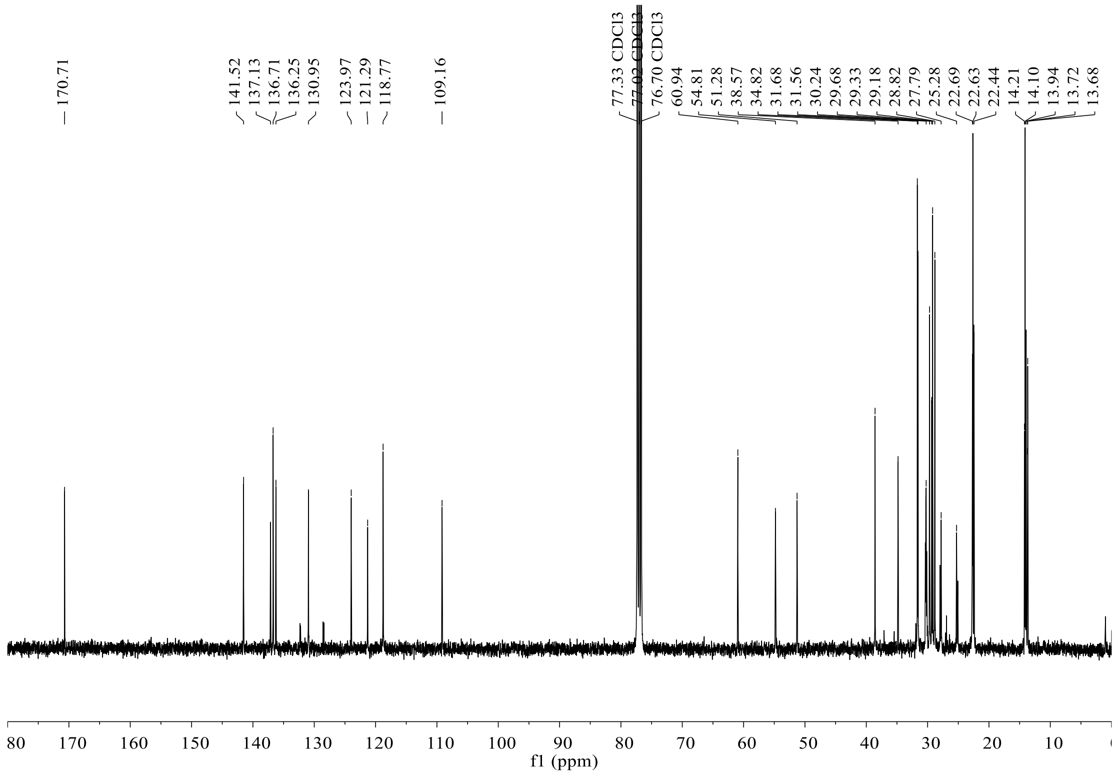

Supplementary Fig. 11 | ^{13}C NMR spectrum of compound E3-4 in $CDCl_3$.

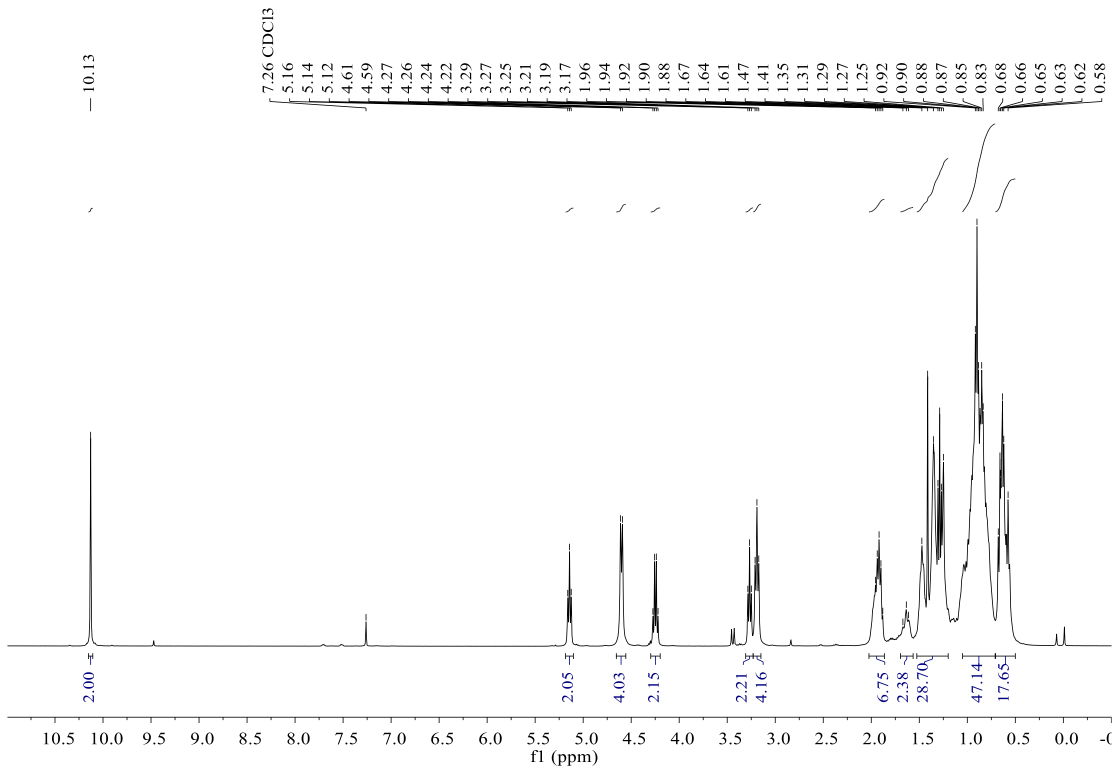

Supplementary Fig. 12 | ^{1}H NMR spectrum of compound E3-5 in $CDCl_3$.

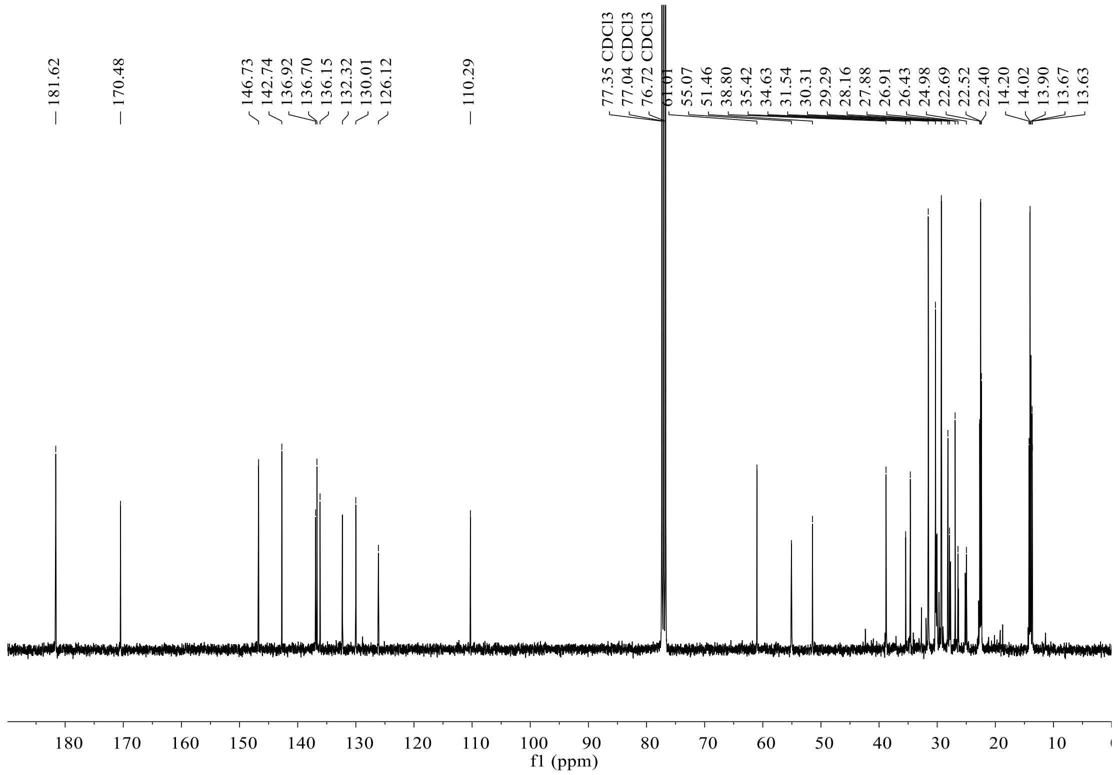

Supplementary Fig. 13 | ^{13}C NMR spectrum of compound E3-5 in $CDCl_3$.

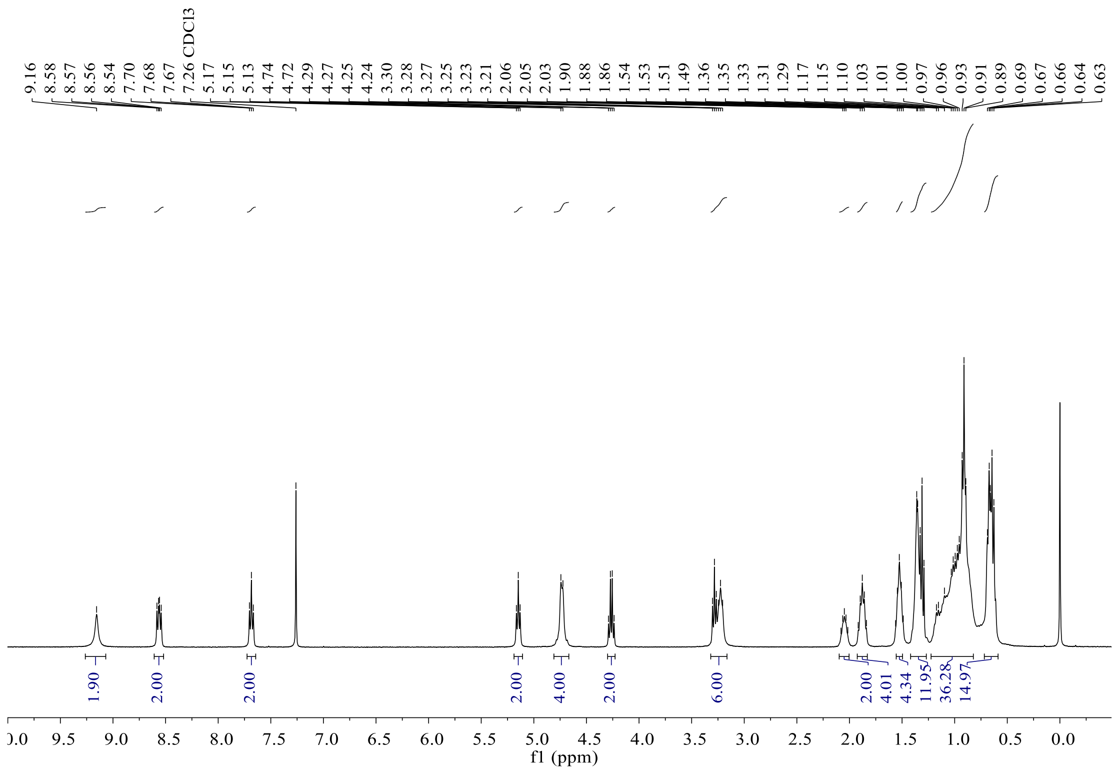

Supplementary Fig. 14 | ^{1}H NMR spectrum of BTZ-E3 in $CDCl_3$.

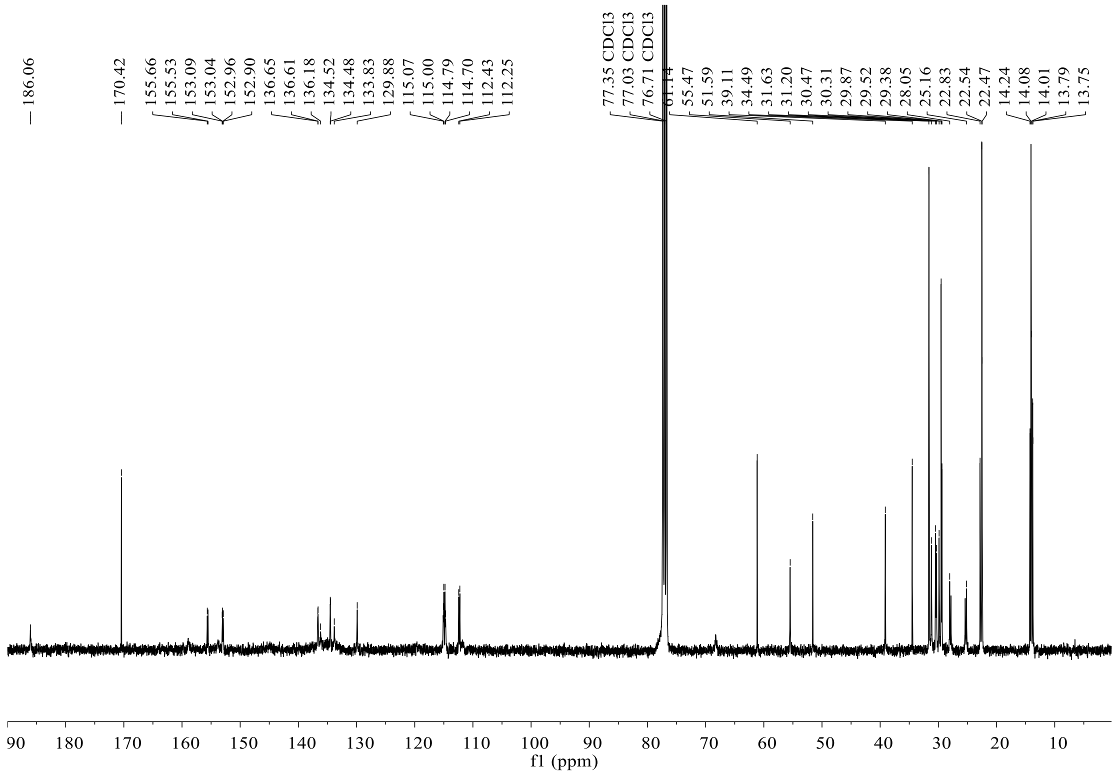

Supplementary Fig. 15 | ^{13}C NMR spectrum of BTZ-E3 in $CDCl_3$.

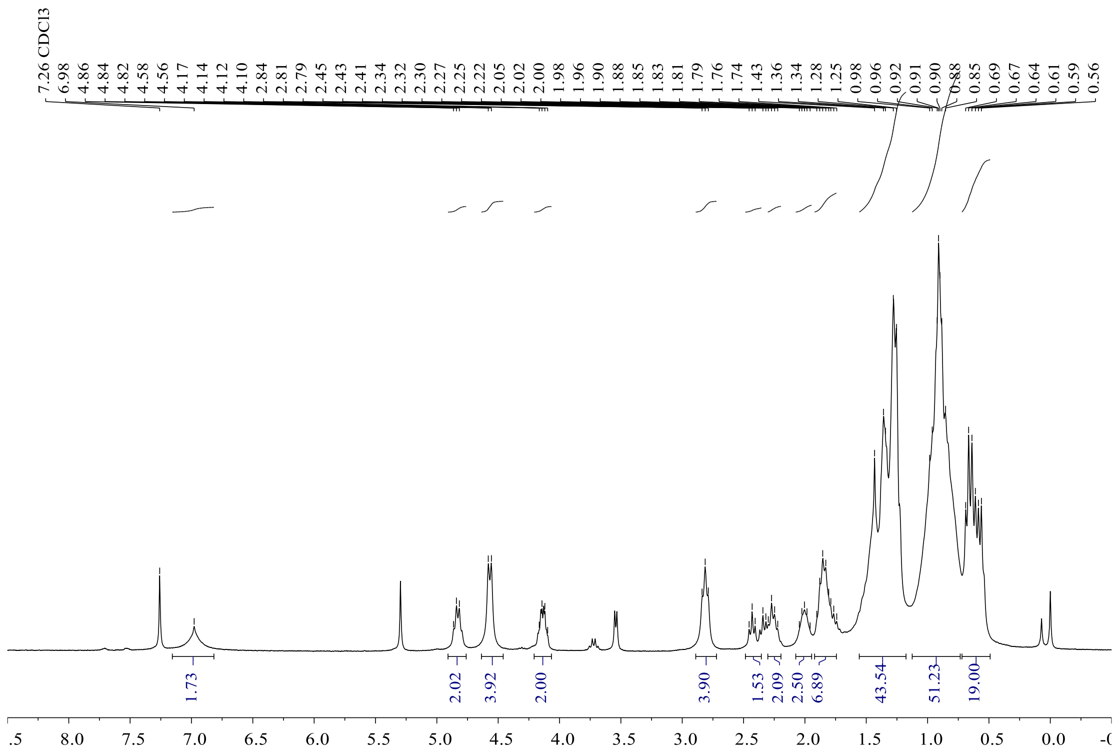

Supplementary Fig. 16 | ^{1}H NMR spectrum of compound E6-2 in $CDCl_3$.

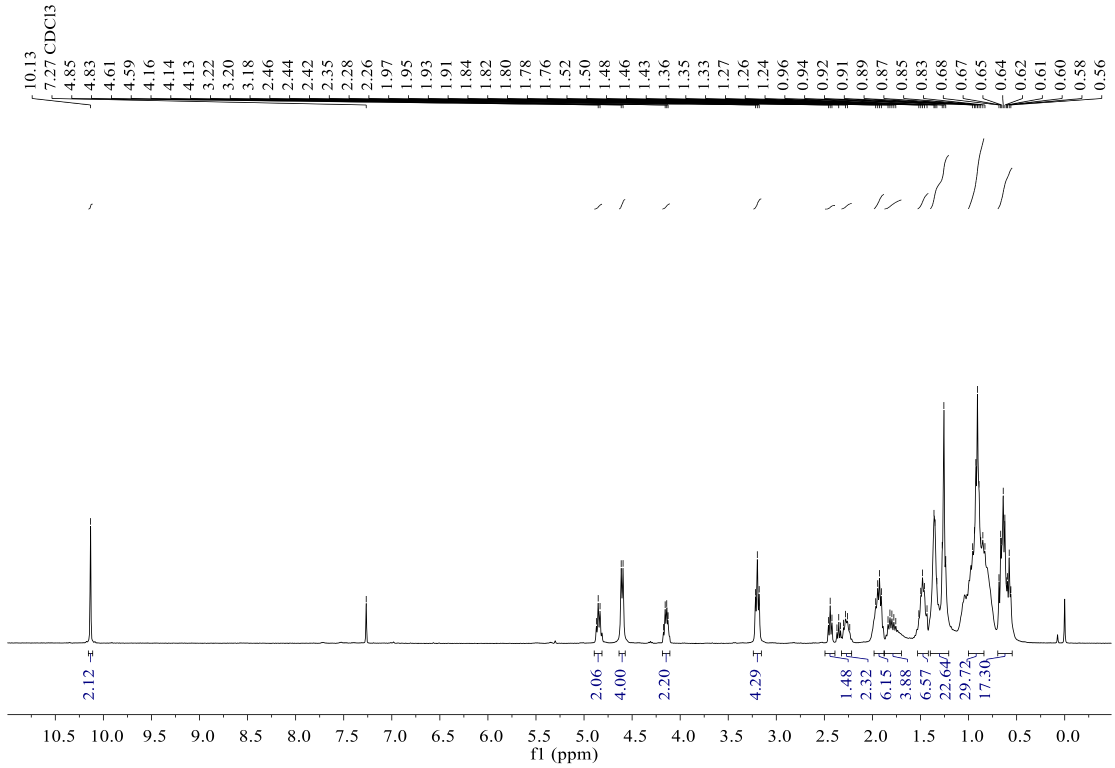

Supplementary Fig. 17 | ^{1}H NMR spectrum of compound E6-3 in $CDCl_3$.

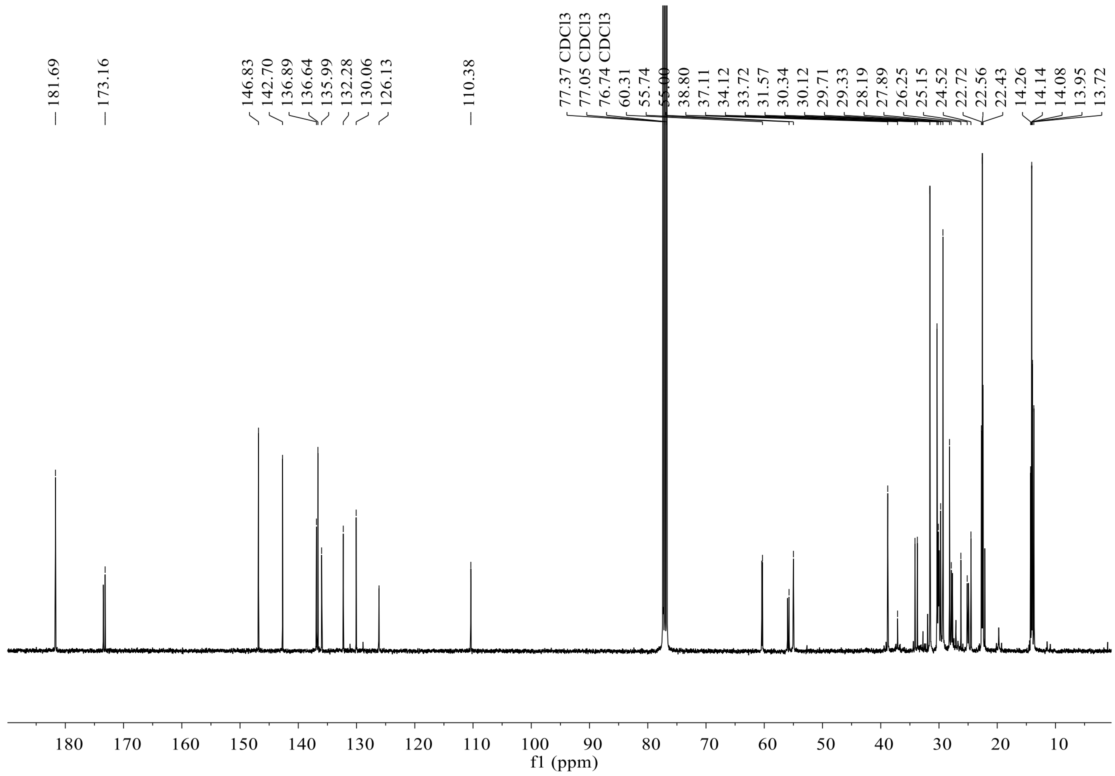

Supplementary Fig. 18 | ^{13}C NMR spectrum of compound E6-3 in $CDCl_3$.

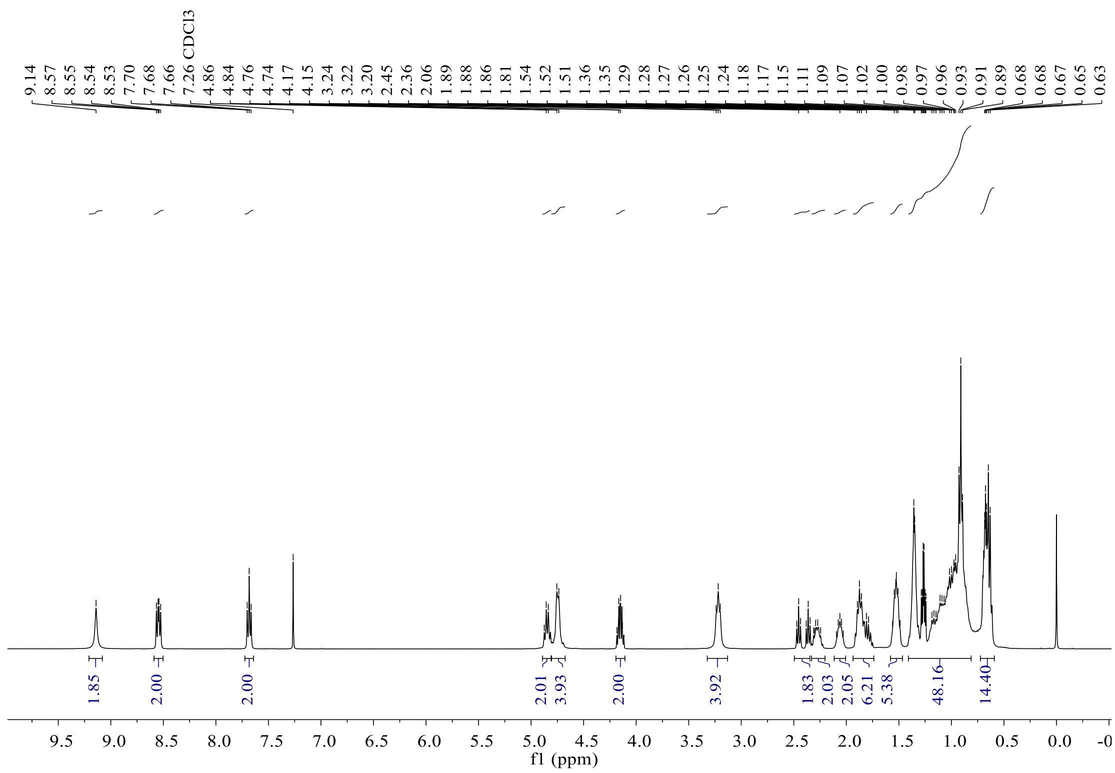

Supplementary Fig. 19 | ^{1}H NMR spectrum of BTZ-E6 in $CDCl_3$.

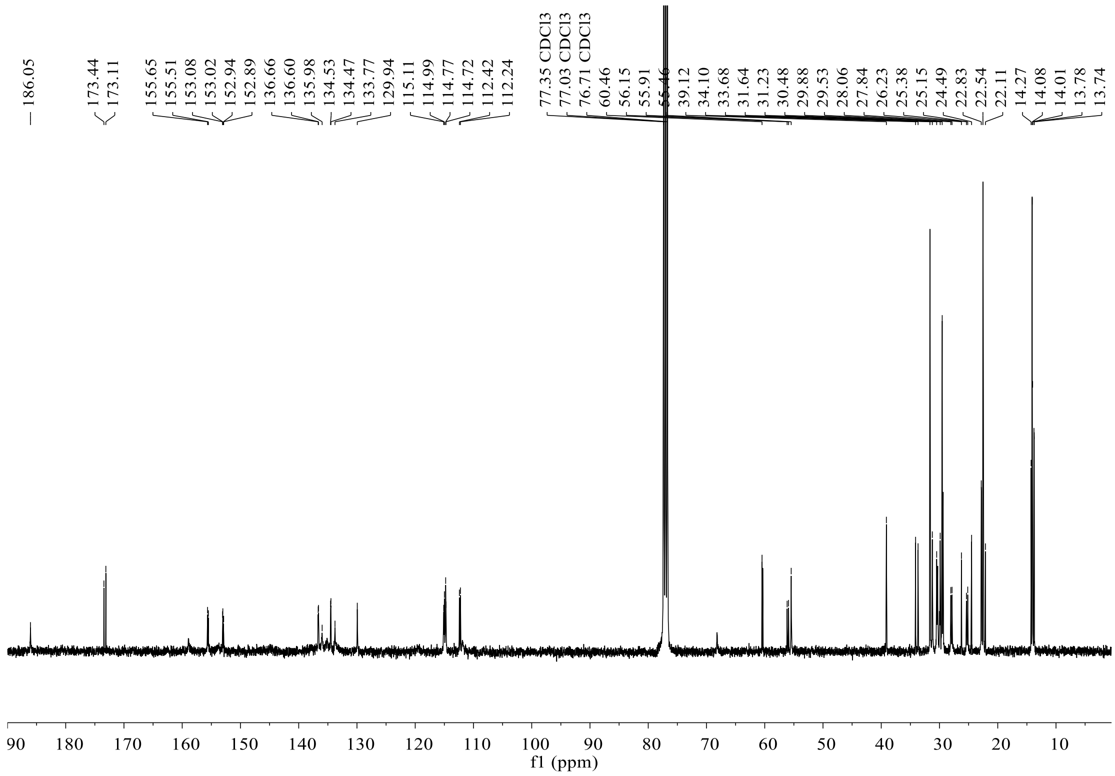

Supplementary Fig. 20 | ^{13}C NMR spectrum of BTZ-E6 in $CDCl_3$.

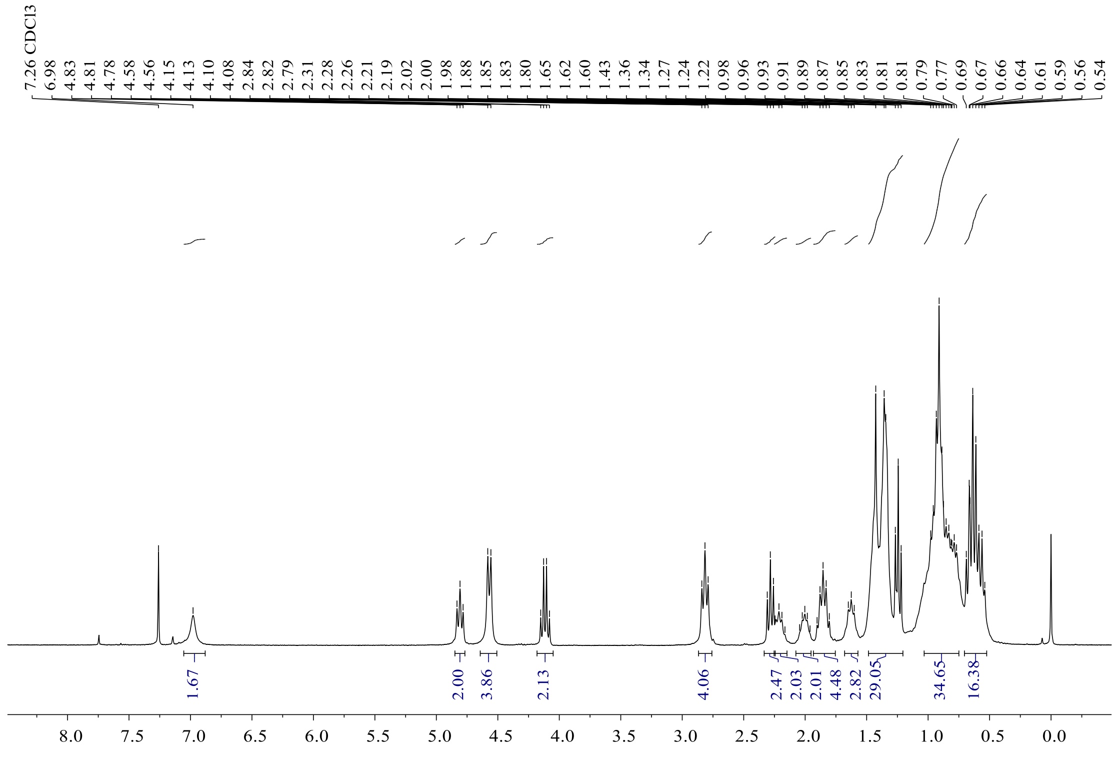

Supplementary Fig. 21 | ^{1}H NMR spectrum of compound E9-2 in $CDCl_3$.

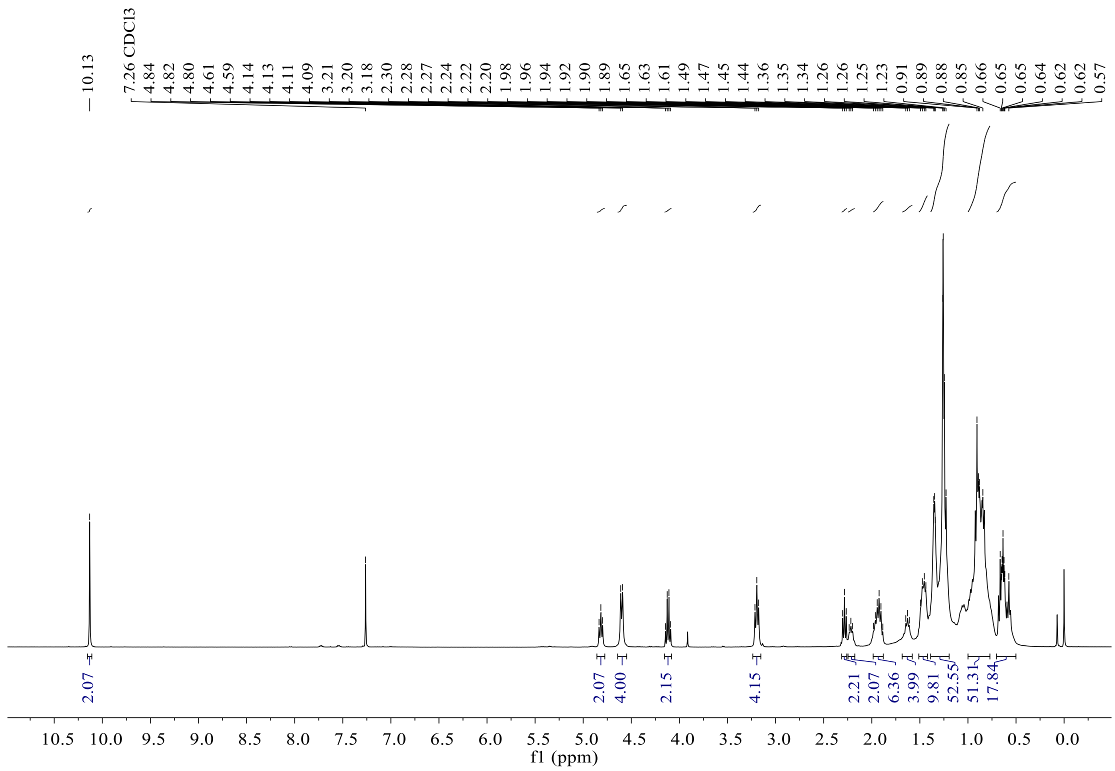

Supplementary Fig. 22 | ^{1}H NMR spectrum of compound E9-3 in $CDCl_3$.

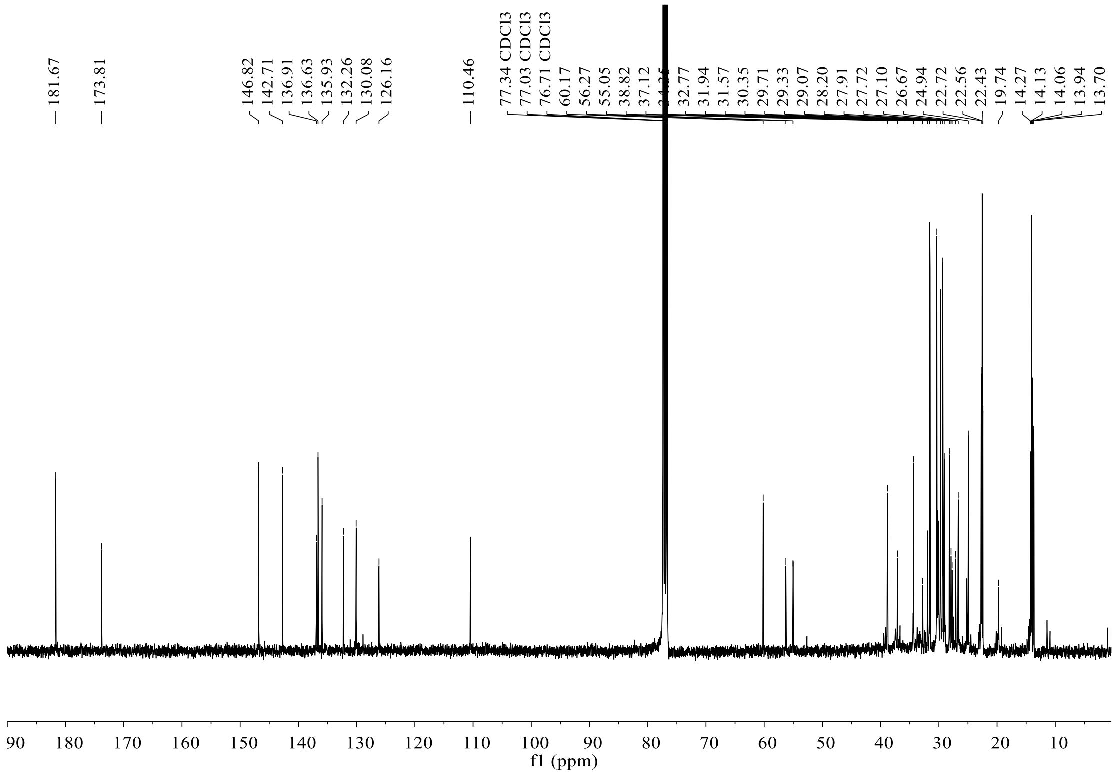

Supplementary Fig. 23 | ^{13}C NMR spectrum of compound E9-3 in $CDCl_3$.

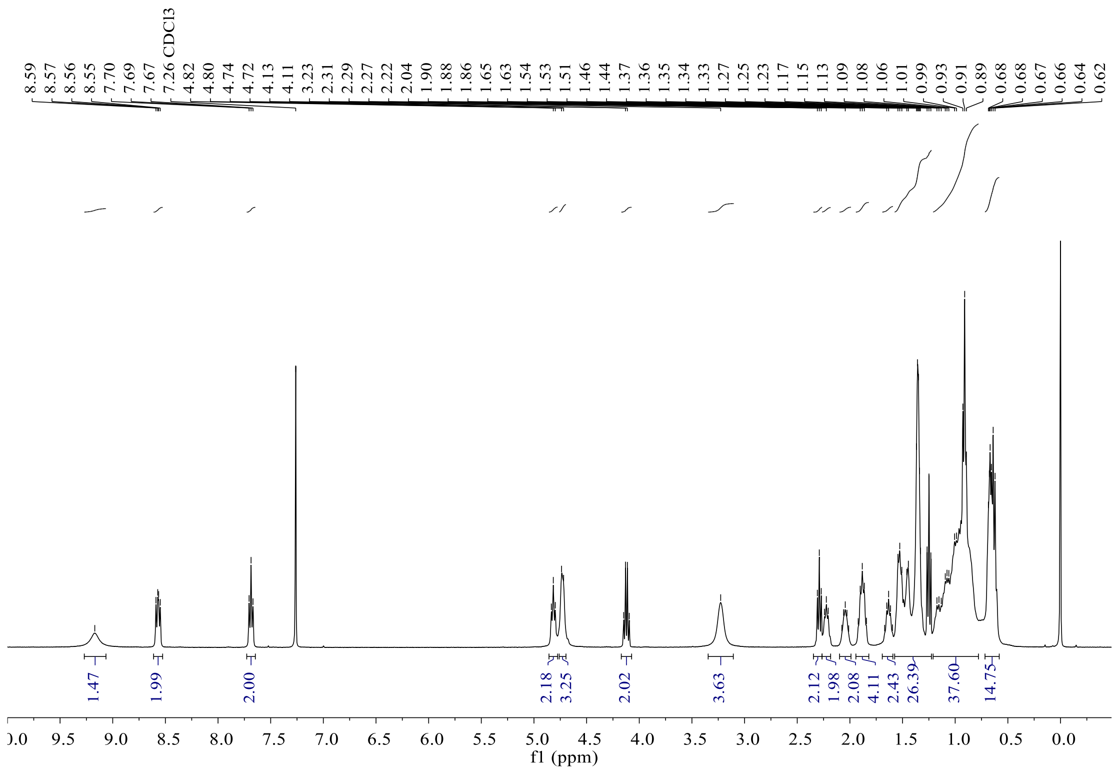

Supplementary Fig. 24 | ^{1}H NMR spectrum of BTZ-E9 in $CDCl_3$.

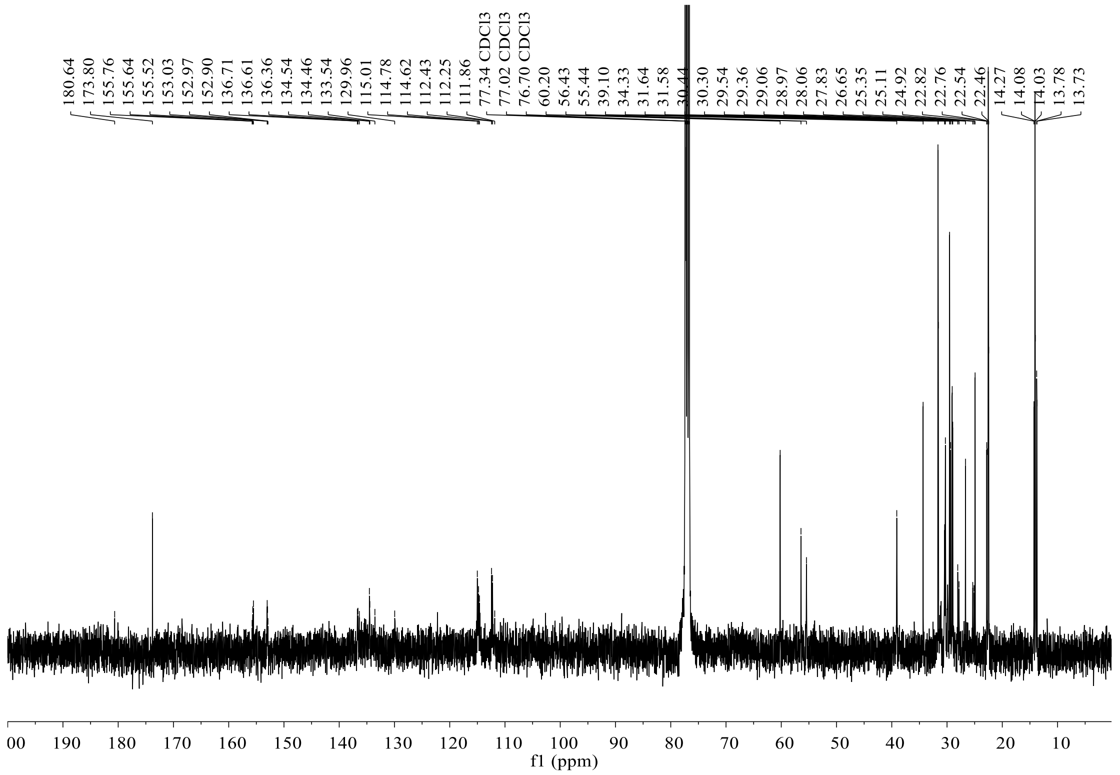

Supplementary Fig. 25 | ^{13}C NMR spectrum of BTZ-E9 in $CDCl_3$.

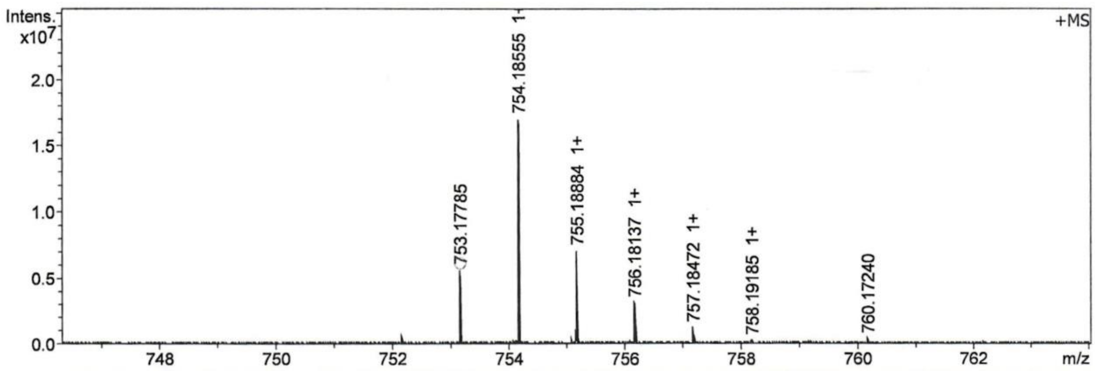

Supplementary Fig. 26 | The mass spectrum (MALDI-TOF) of compound E3-3.

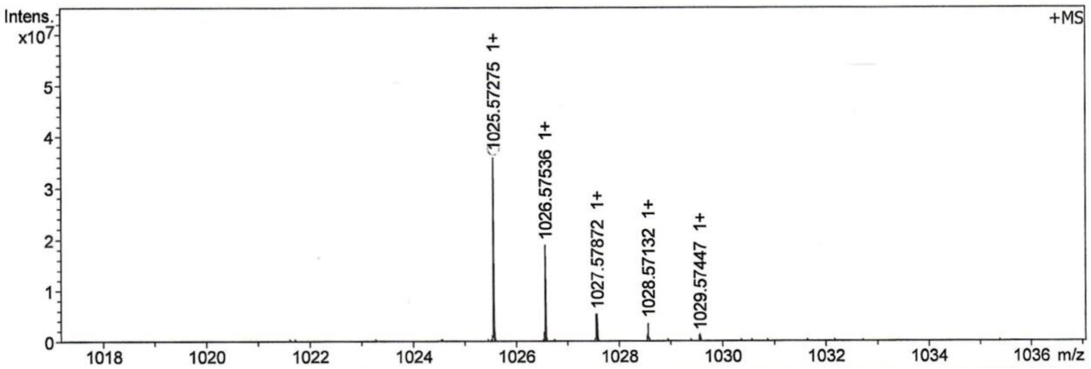

Supplementary Fig. 27 | The mass spectrum (MALDI-TOF) of compound E3-4.

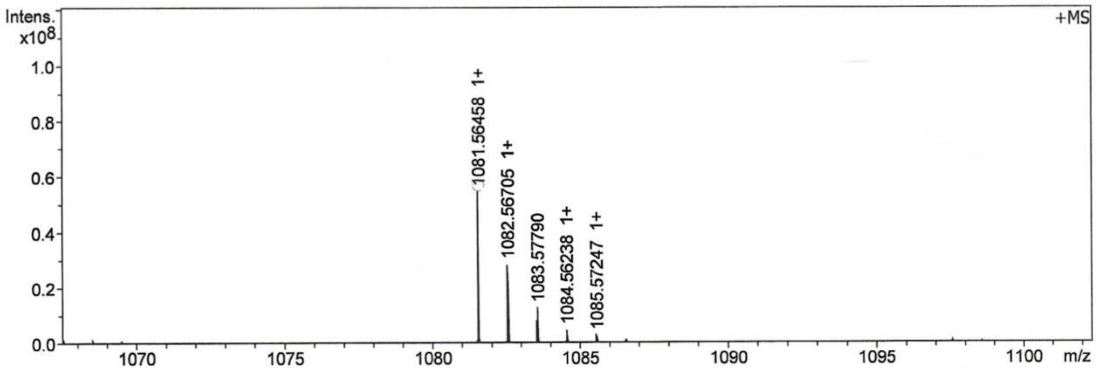

Supplementary Fig. 28 | The mass spectrum (MALDI-TOF) of compound E3-5.

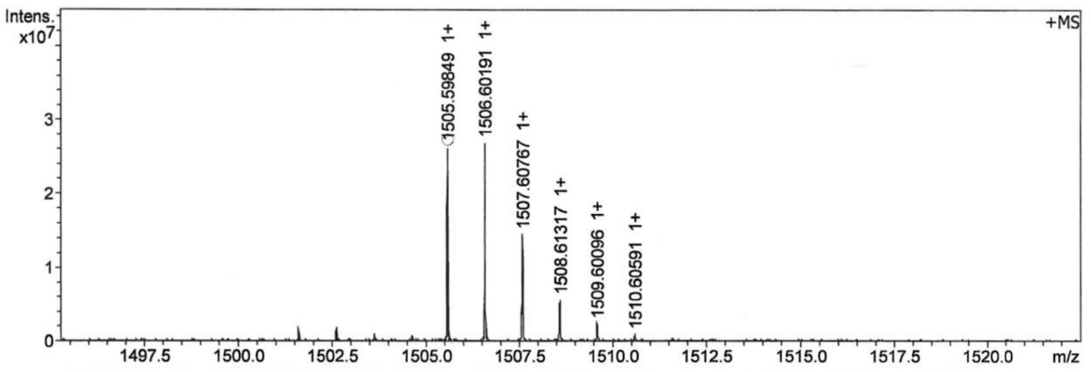

Supplementary Fig. 29 | The mass spectrum (MALDI-TOF) of BTZ-E3.

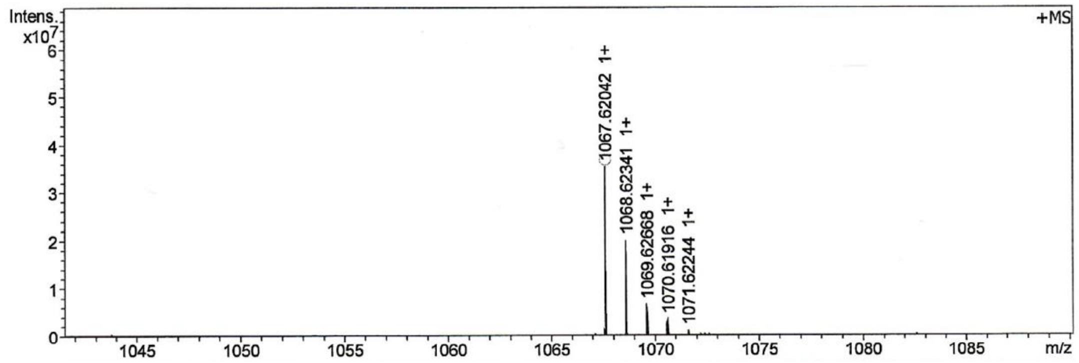

Supplementary Fig. 30 | The mass spectrum (MALDI-TOF) of E6-2.

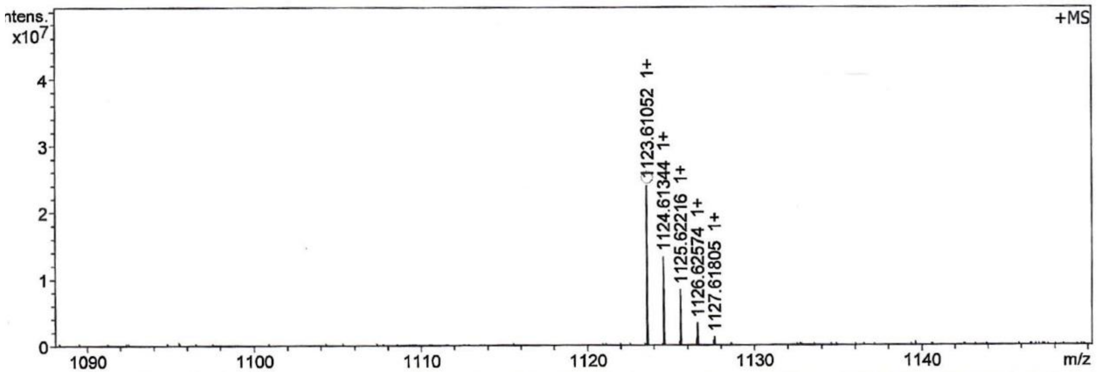

Supplementary Fig. 31 | The mass spectrum (MALDI-TOF) of E6-3.

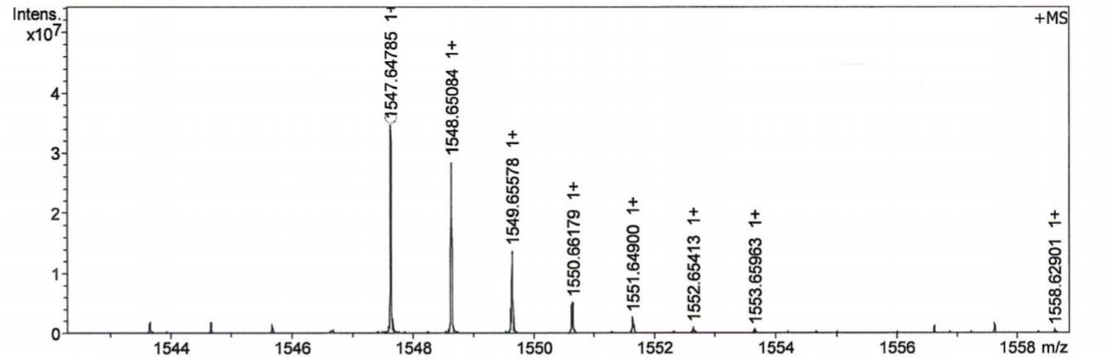

Supplementary Fig. 32 | The mass spectrum (MALDI-TOF) of BTZ-E6.

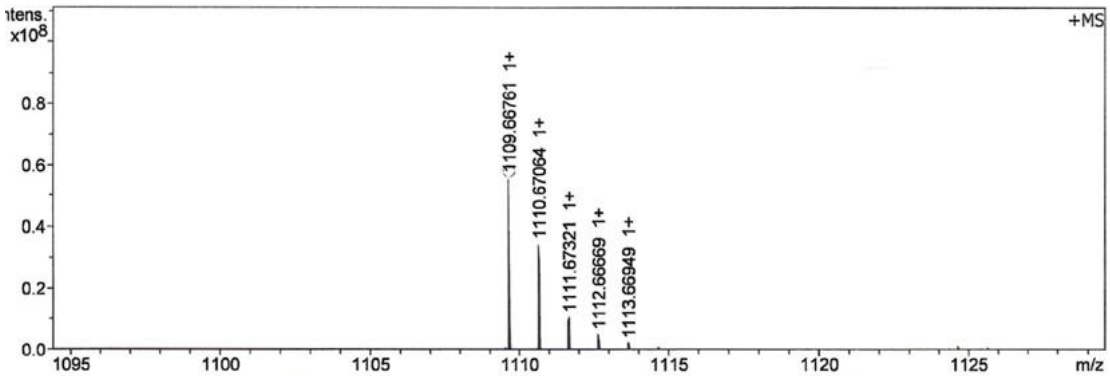

Supplementary Fig. 33 | The mass spectrum (MALDI-TOF) of E9-2.

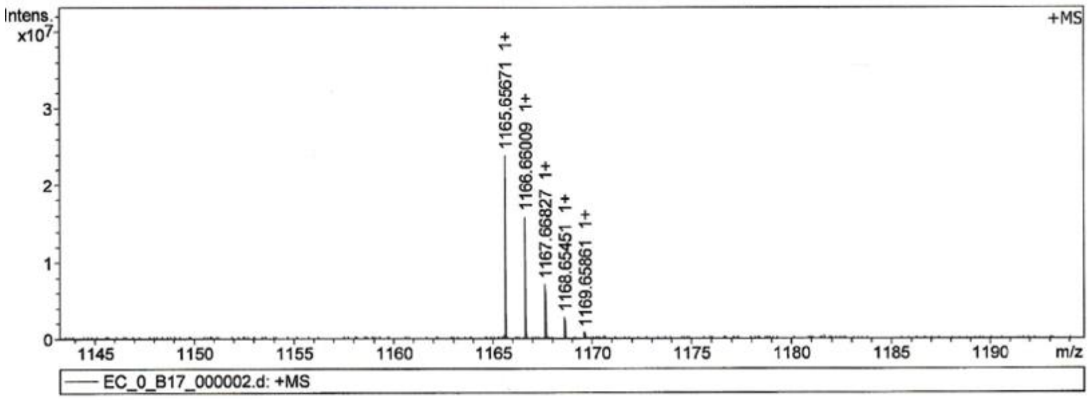

Supplementary Fig. 34 | The mass spectrum (MALDI-TOF) of E9-3.

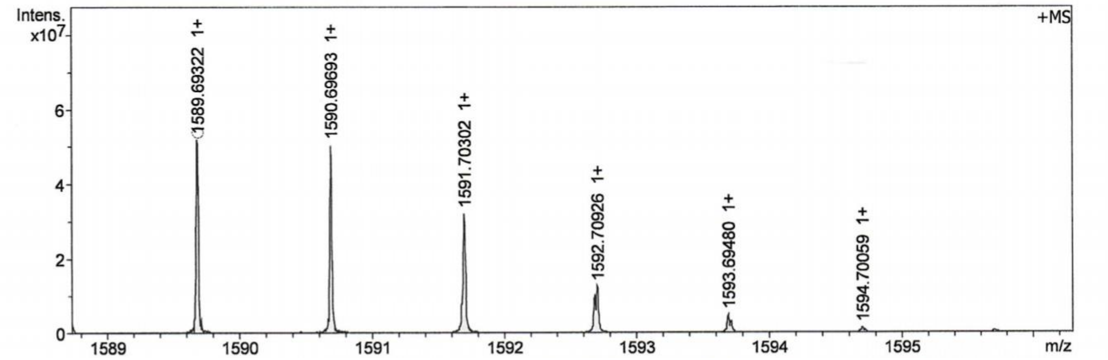

Supplementary Fig. 35 | The mass spectrum (MALDI-TOF) of BTZ-E9.

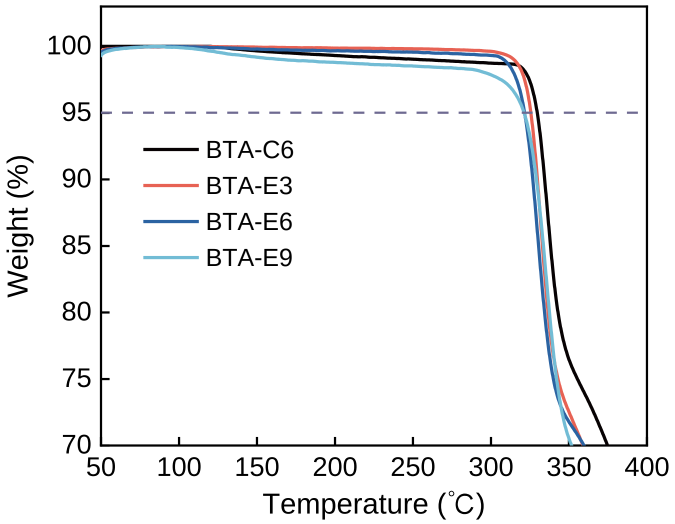

Supplementary Fig. 36 | TGA curves of BTA-C6, BTA-E3, BTA-E6 and BTA-E9.

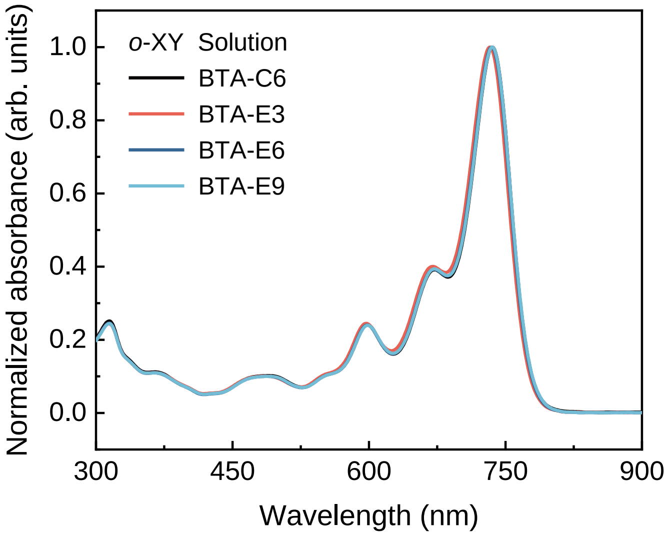

Supplementary Fig. 37 | UV-vis absorption spectra of BTA-C6, BTA-E3, BTA-E6 and BTA-E9 in *o*-xylene solutions.

Source data are provided as a Source Data file.

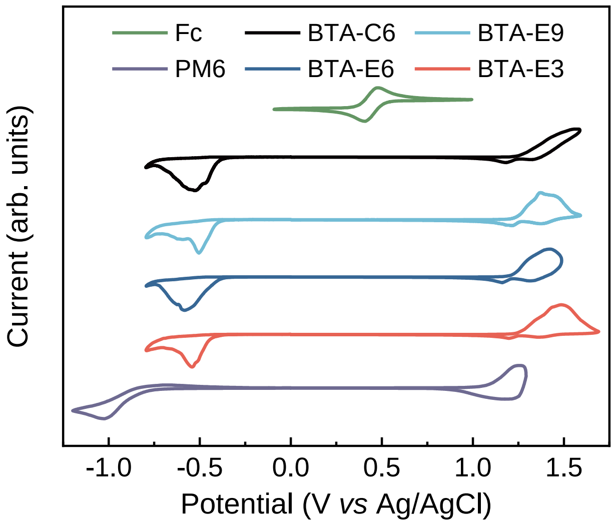

Supplementary Fig. 38 | The cyclic voltammetry curves of PM6, BTA-C6, BTA-E3, BTA-E6 and BTA-E9.

c

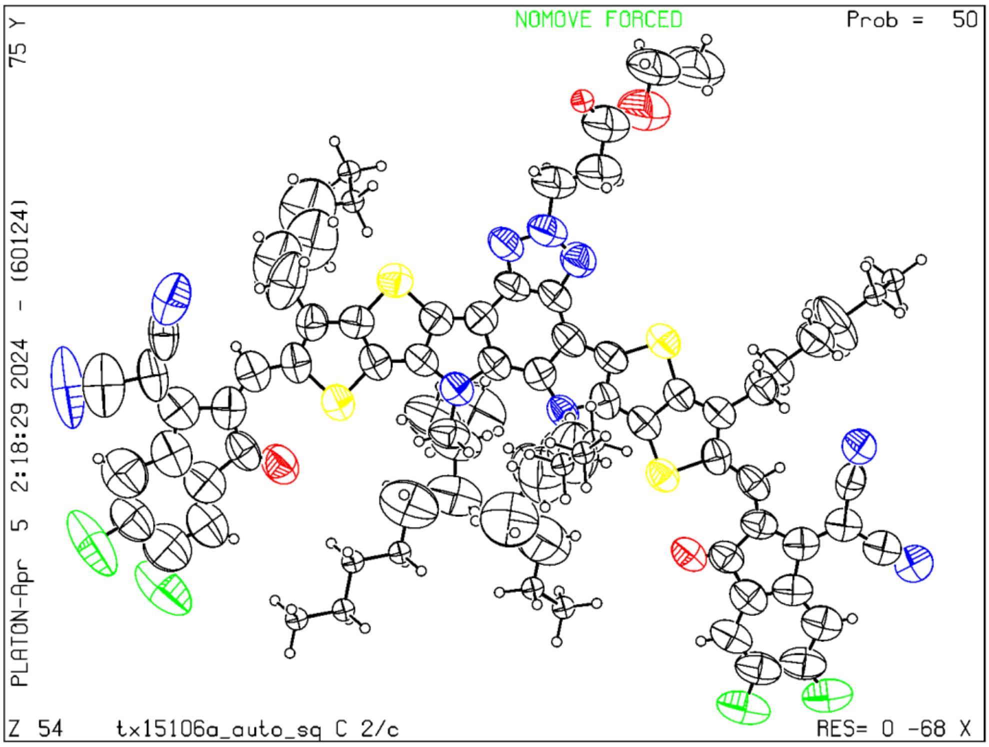

Supplementary Fig. 39 | The Oak Ridge Thermal Ellipsoid Plot (ORTEP) view of BTA-E3 (CCDC number: 2333735) with atomic displacement parameters shown at the 50% probability level. Alerts justification: Numerous crystals obtained from various methods have been attempted, but the quality is still unsatisfactory, which can be attributed to the high degree of disorder caused by outer/inner alkyl chains and the crystal decomposition during the measurement process. Despite the long exposure time, the crystal did not exhibit observable intensity at a resolution above 0.90 angstrom, therefore level A and level B alarms were displayed in the CheckCIF report. However, in this work, we only analyzed based on the Conjugated skeleton and N-alkyl chain of the molecule, not on the outer/inner alkyl chains.

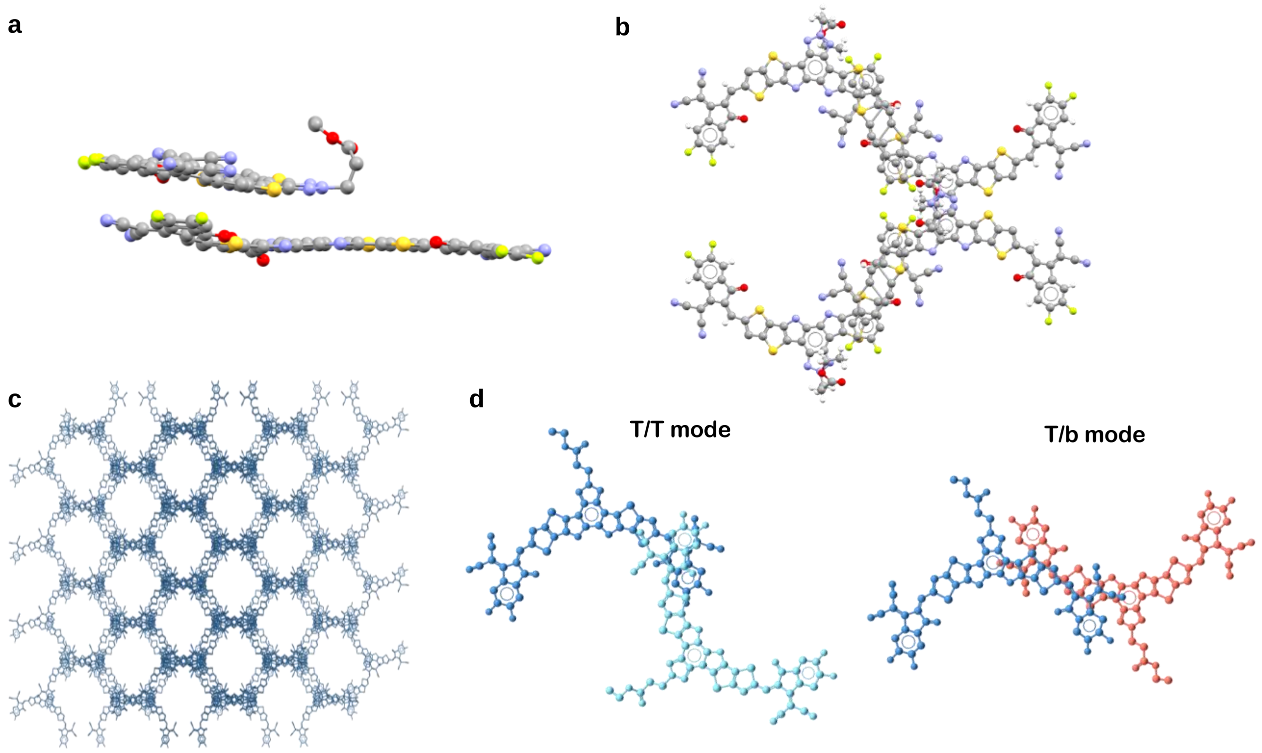

Supplementary Fig. 40 | Single-crystal structure and stacking modes. a, Two different orientations of ethyl ester side chain in BTA-E3. **b,** The single-crystal stacking diagrams in T/b stacking mode of the BTA-E3. **c,** 3D single-crystal stacking diagram of BTA-E3. **d,** Different intermolecular stacking modes of BTA-E3.

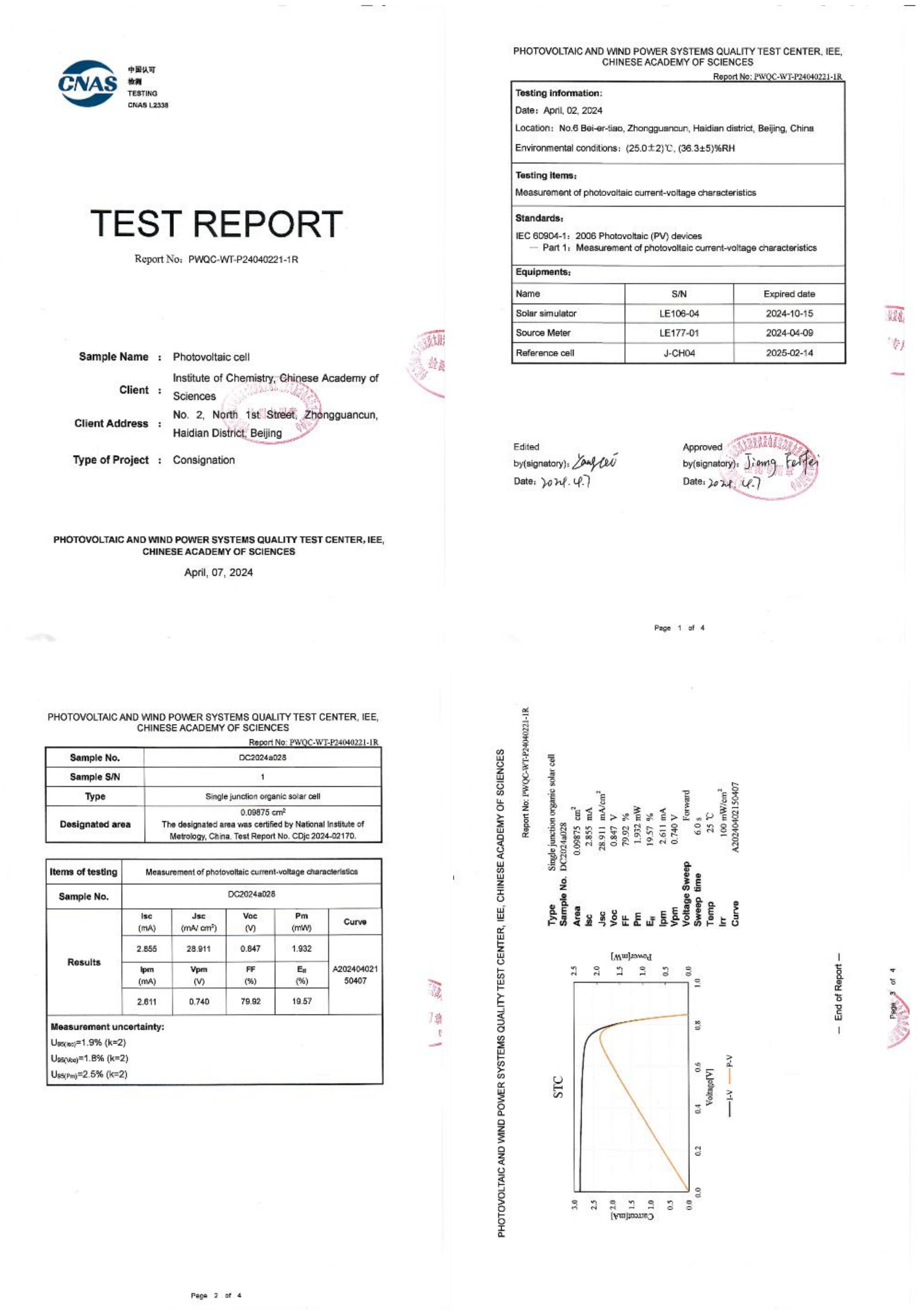
CNAS 中国认可 检测 TESTING CNAS L2338

TEST REPORT

Report No：PWQC-WT-P24040221-1R

Sample Name	:	Photovoltaic cell
Client	:	Institute of Chemistry, Chinese Academy of Sciences
Client Address	:	No. 2, North 1st Street, Zhongguancun, Haidian District, Beijing
Type of Project	:	Consignation

PHOTOVOLTAIC AND WIND POWER SYSTEMS QUALITY TEST CENTER, IEE, CHINESE ACADEMY OF SCIENCES

April, 07, 2024

PHOTOVOLTAIC AND WIND POWER SYSTEMS QUALITY TEST CENTER, IEE, CHINESE ACADEMY OF SCIENCES

Report No: PWQC-WT-P24040221-1R

Testing information:

Date：April, 02, 2024

Location：No.6 Bei-er-tiao, Zhongguancun, Haidian district, Beijing, China

Environmental conditions：(25.0±2)℃, (36.3±5)%RH

Testing items:

Measurement of photovoltaic current-voltage characteristics

Standards:

IEC 60904-1：2006 Photovoltaic (PV) devices
— Part 1：Measurement of photovoltaic current-voltage characteristics

Equipments:

Name	S/N	Expired date
Solar simulator	LE106-04	2024-10-15
Source Meter	LE177-01	2024-04-09
Reference cell	J-CH04	2025-02-14

Edited by(signatory): Zangcai
Date：2024.4.7

Approved by(signatory): Jiang Feifei
Date：2024.4.7

Page 1 of 4

PHOTOVOLTAIC AND WIND POWER SYSTEMS QUALITY TEST CENTER, IEE, CHINESE ACADEMY OF SCIENCES

Report No: PWQC-WT-P24040221-1R

Sample No.	DC2024a028
Sample S/N	1
Type	Single junction organic solar cell
Designated area	0.09875 cm^2 The designated area was certified by National Institute of Metrology, China. Test Report No. CDjc 2024-02170.

Items of testing	Measurement of photovoltaic current-voltage characteristics				
Sample No.	DC2024a028				
Results	Isc (mA)	Jsc (mA/cm^2)	Voc (V)	Pm (mW)	Curve
	2.855	28.911	0.847	1.932	A20240402150407
	Ipm (mA)	Vpm (V)	FF (%)	E_{ff} (%)	
	2.611	0.740	79.92	19.57	

Measurement uncertainty:

$U_{95(Isc)}$=1.9% (k=2)
$U_{95(Voc)}$=1.8% (k=2)
$U_{95(Pm)}$=2.5% (k=2)

Page 2 of 4

PHOTOVOLTAIC AND WIND POWER SYSTEMS QUALITY TEST CENTER, IEE, CHINESE ACADEMY OF SCIENCES

Report No: PWQC-WT-P24040221-1R

Type	Single junction organic solar cell
Sample No.	DC2024a028
Area	0.09875 cm^2
Isc	2.855 mA
Jsc	28.911 mA/cm^2
Voc	0.847 V
FF	79.92 %
Pm	1.932 mW
E_{ff}	19.57 %
Ipm	2.611 mA
Vpm	0.740 V
Voltage Sweep	Forward
Sweep time	6.0 s
Temp	25 ℃
Irr	100 mW/cm^2
Curve	A20240402150407

— End of Report —

Page 3 of 4

Supplementary Fig. 41 | Original report of the OSC certificate results by the Photovoltaic and Wind Power Systems Quality Test Center, IEE, Chinese Academy of Sciences.

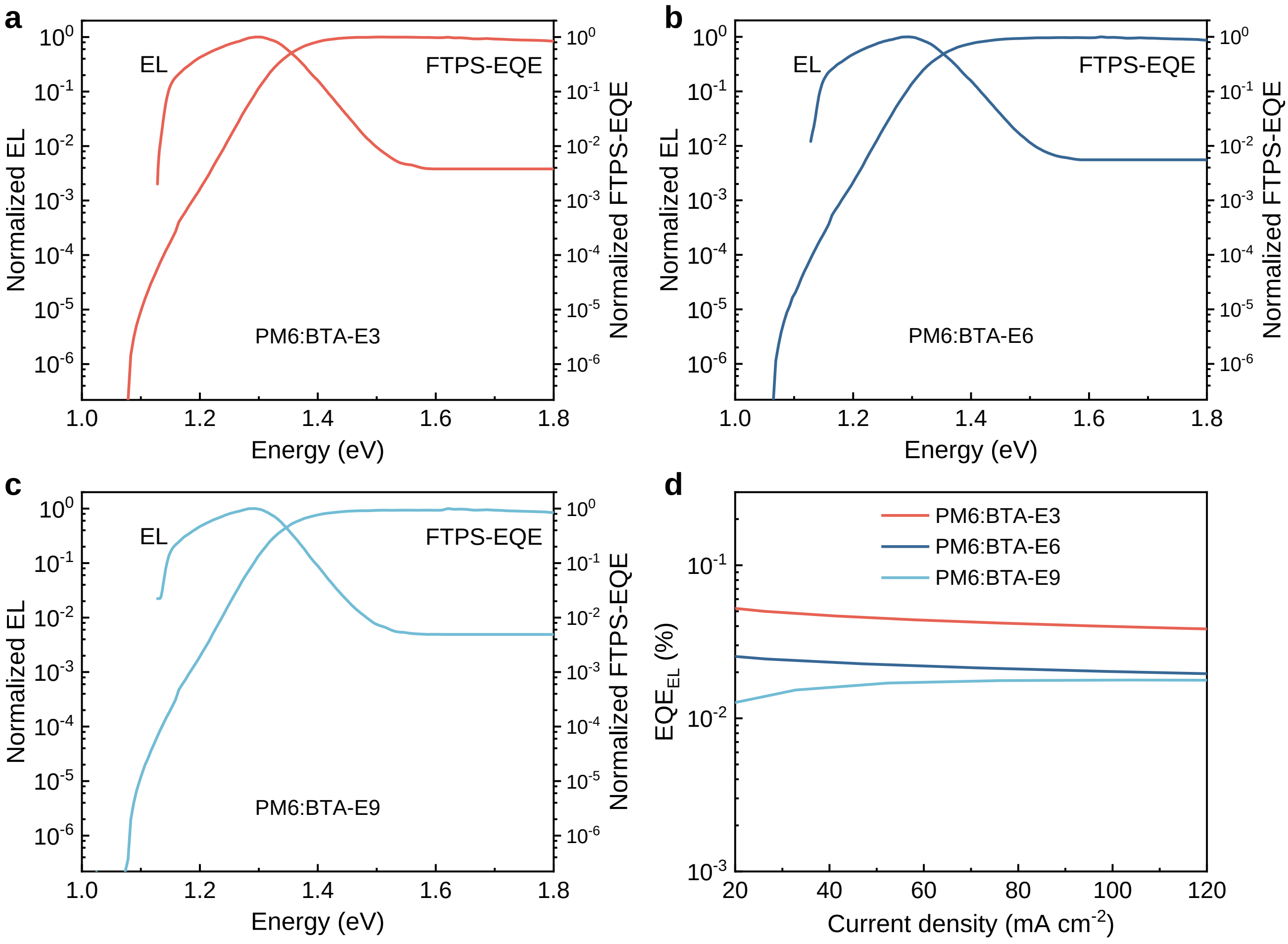

Supplementary Fig. 42 | Energy loss measurement results. a-c, Semilogarithmic plots of normalized EL and normalized FTPS-EQE of PM6:BTA-E3 (**a**), PM6:BTA-E6 (**b**) and PM6:BTA-E9 (**c**) based devices. **d,** The EQE_{EL} of PM6:BTA-E3, PM6:BTA-E6 and PM6:BTA-E9 based devices at different injected currents.

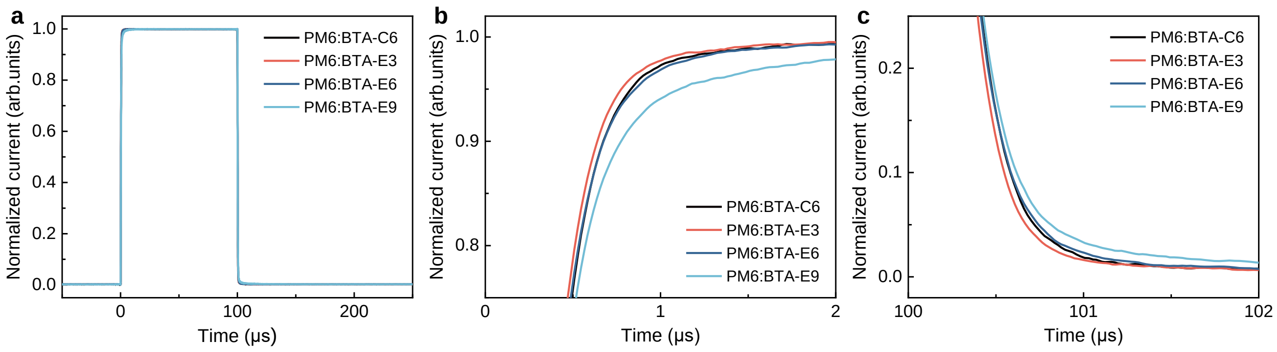

Supplementary Fig. 43 | TPC measurement. **a,** Normalized transient photocurrents in response to a 100 μs white light (LED) pulse for the devices. **b,c,** Rise (**b**) and decay (**c**) curves of the photocurrent in the TPC test with time.

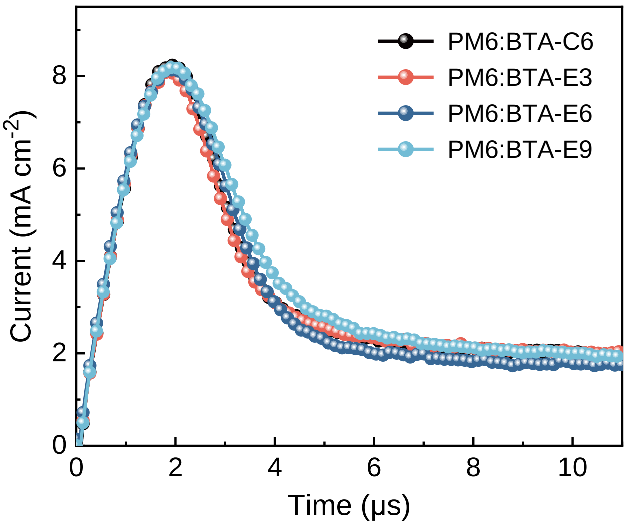

Supplementary Fig. 44 | Photo-CELIV curves of the OSCs based on PM6:BTA-C6, PM6:BTA-E3, PM6:BTA-E6 and PM6:BTA-E9.

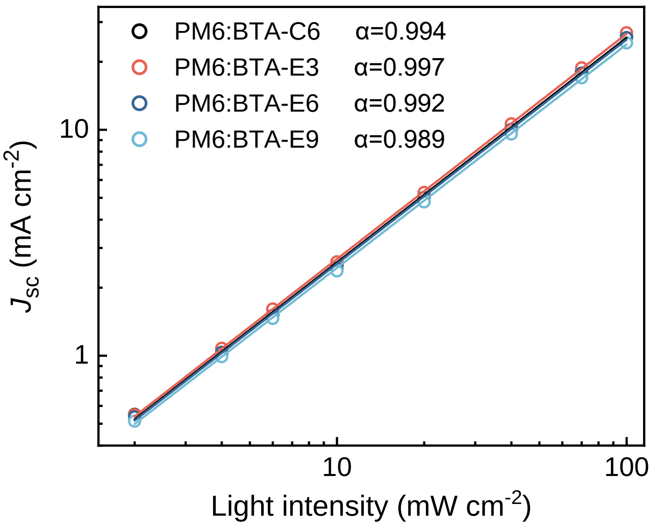

Supplementary Fig. 45 | The dependence of J_{sc} on light intensity of the PM6:BTA-C6, PM6:BTA-E3, PM6:BTA-E6 and PM6:BTA-E9 based devices. Source data are provided as a Source Data file.

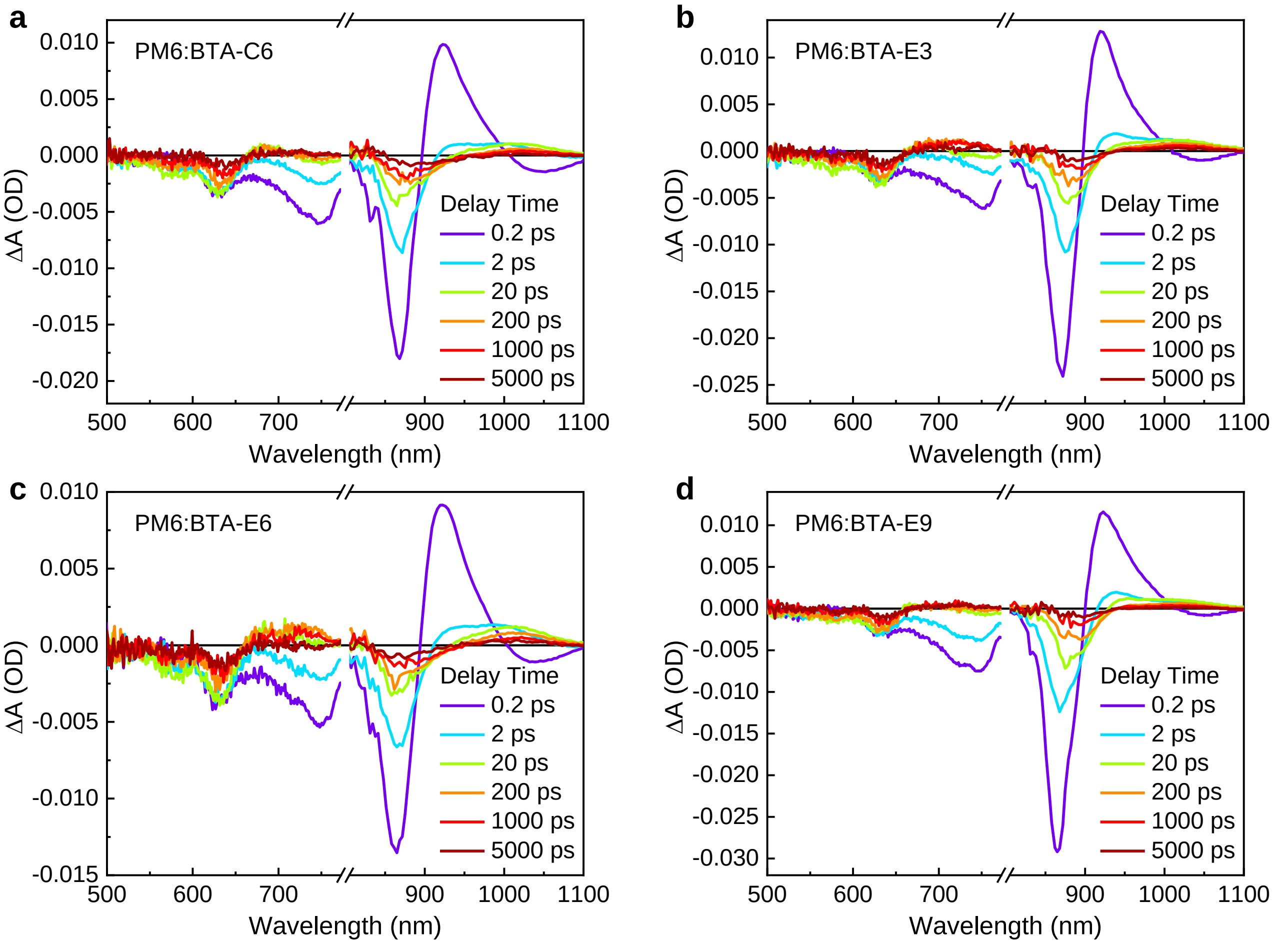

Supplementary Fig. 46 | TA measurement. a-d, Femtosecond transient absorption spectra of PM6:BTA-C6 (**a**), PM6:BTA-E3 (**b**), PM6:BTA-E6 (**c**) and PM6:BTA-E9 (**d**) blend films at the selected delay times.

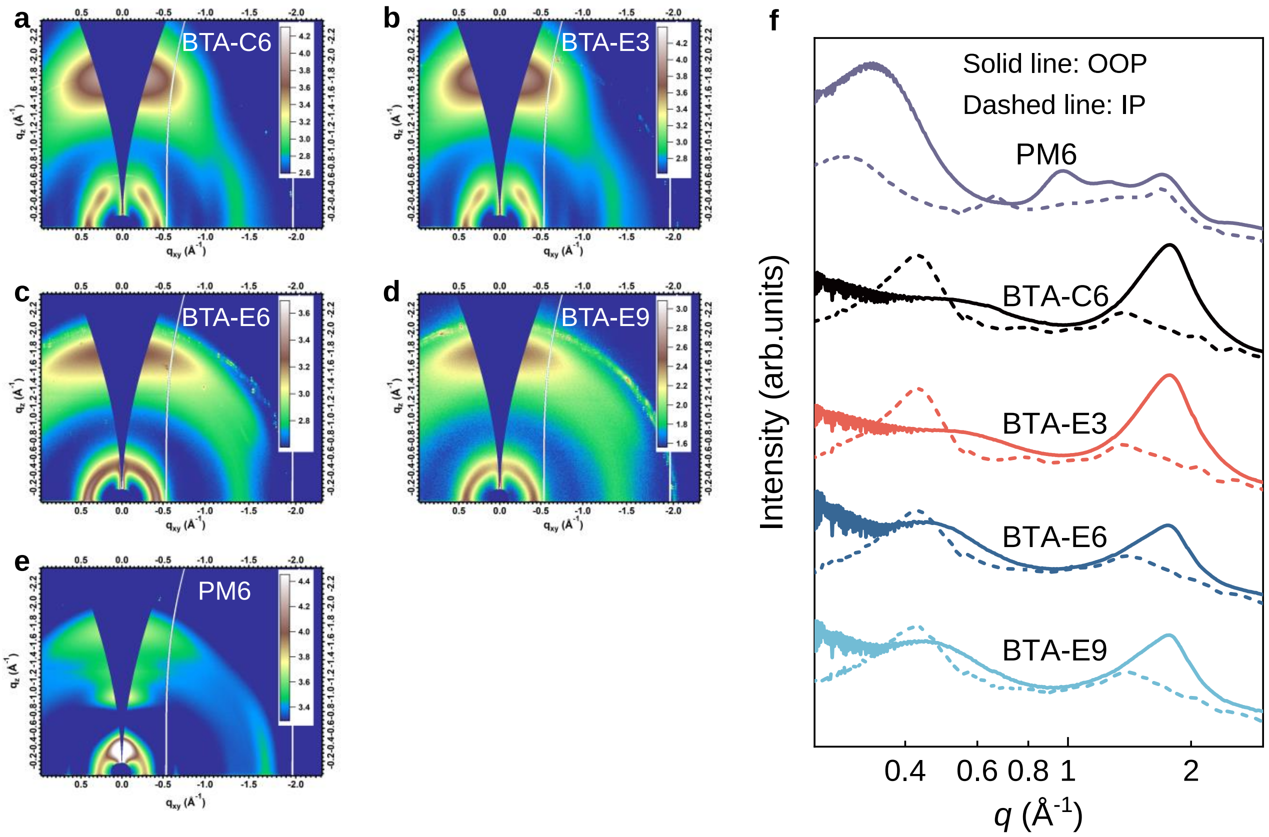

Supplementary Fig. 47 | GIWAXS measurements. **a-e,** 2D GIWAXS patterns of BTA-C6 (**a**), BTA-E3 (**b**), BTA-E6 (**c**), BTA-E9 (**d**) and PM6 (**e**) neat films. **f,** IP and OOP 1D line cut profiles of the 2D GIWAXS data based on the corresponding neat films.

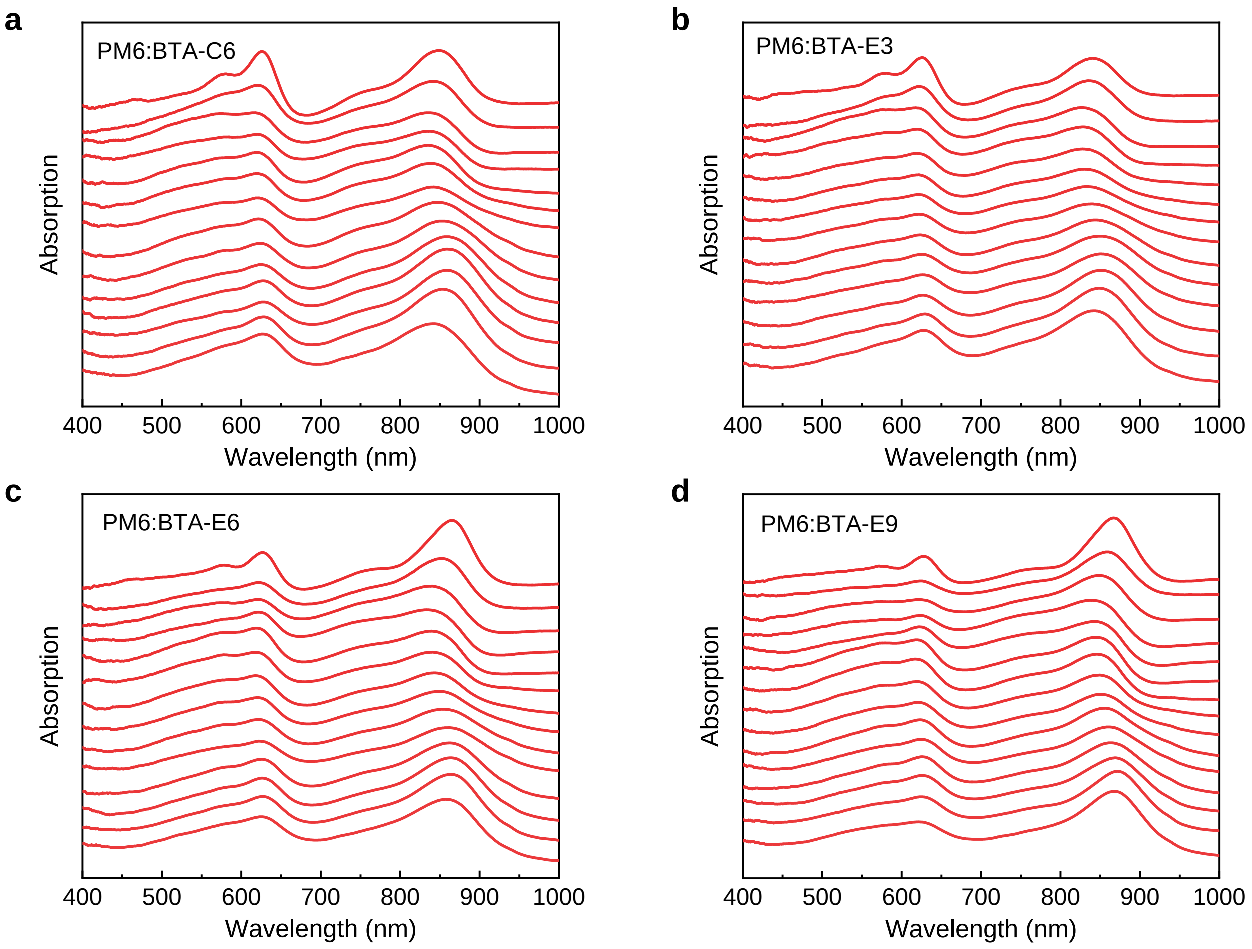

Supplementary Fig. 48 | FLAS measurement. **a-d,** Absorption spectra of sublayers of PM6:BTA-C6 (**a**), PM6:BTA-E3 (**b**), PM6:BTA-E6 (**c**) and PM6:BTA-E9 (**d**) blend films obtained from FLAS.

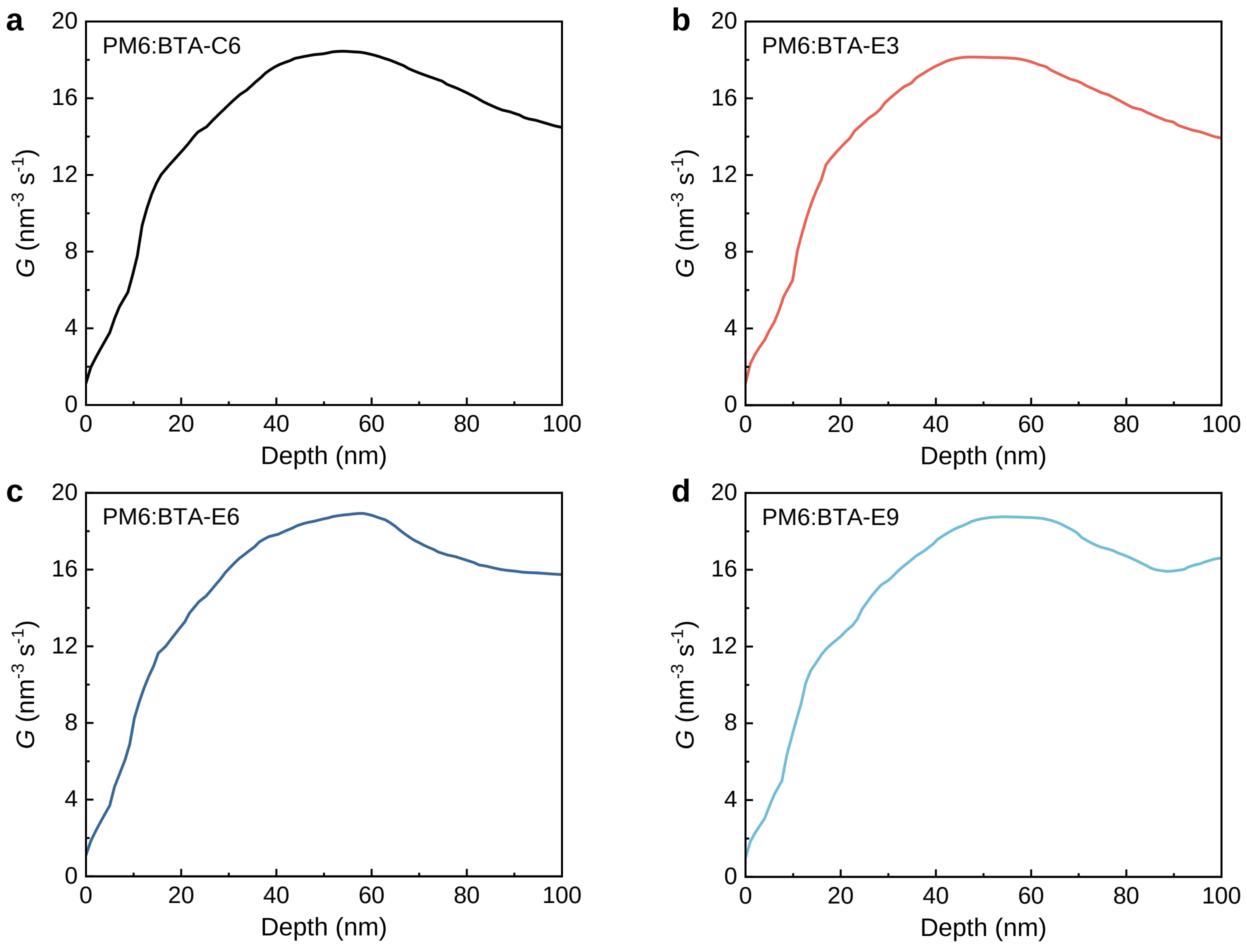

Supplementary Fig. 49 | Exciton generation rate simulation. **a-d,** Dependence of the simulated exciton generation rate (*G*) on the film depth of PM6:BTA-C6 (**a**), PM6:BTA-E3 (**b**), PM6:BTA-E6 (**c**) and PM6:BTA-E9 (**d**) blend films.

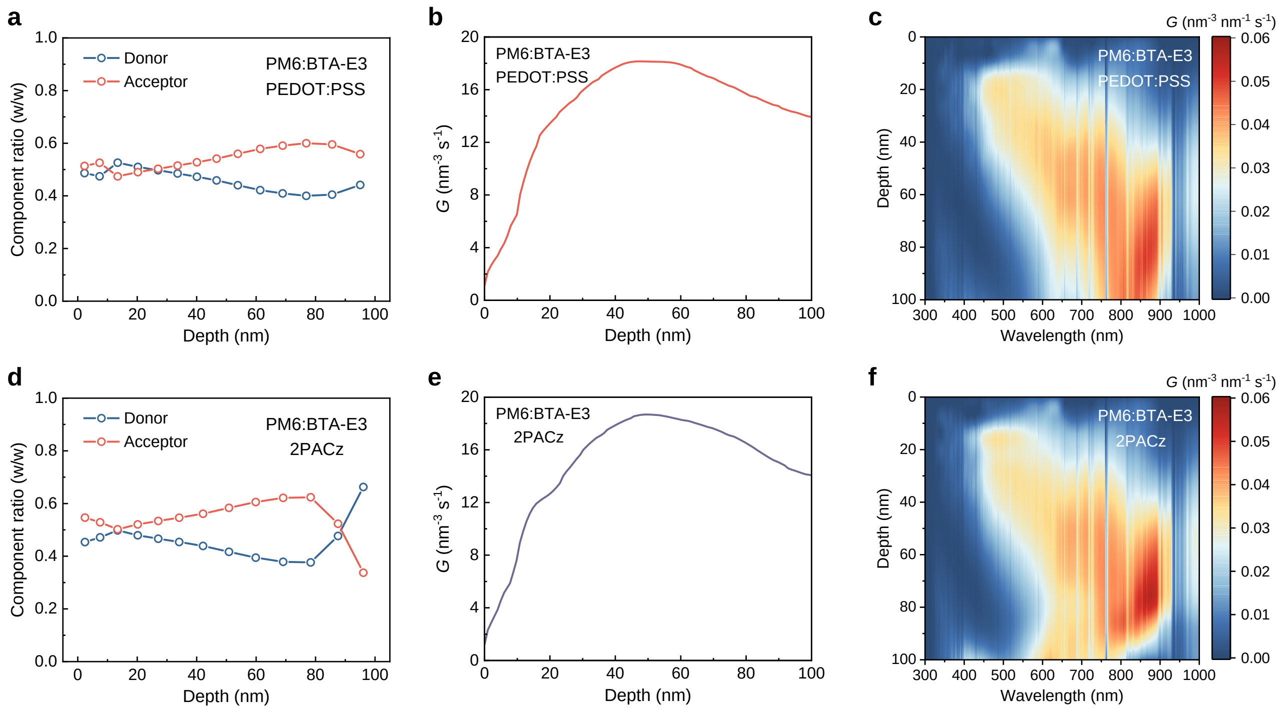

Supplementary Fig. 50 | Vertical phase distribution. **a,d,** Components distribution profiles of the PM6:BTA-E3 blend films on PEDOT:PSS (**a**) and 2PACz (**d**) substrate at different film-depths. **b,e,** Dependence of the simulated exciton generation rate (*G*) on the film depth of PM6:BTA-E3 blend films on PEDOT:PSS (**b**) and 2PACz (**e**) substrate. **c,f,** Numerical simulations for the exciton generation contours of PM6:BTA-E3 blend films on PEDOT:PSS (**c**) and 2PACz (**f**) substrate.

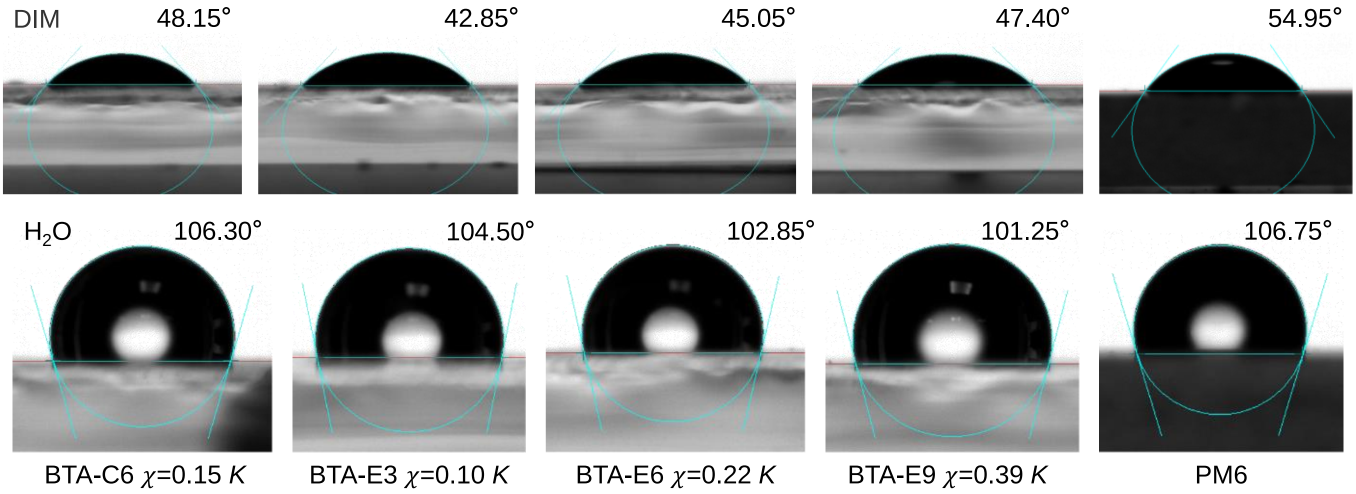

Supplementary Fig. 51 | Contact angle of BTA-C6, BTA-E3, BTA-E6, BTA-E9 and PM6.

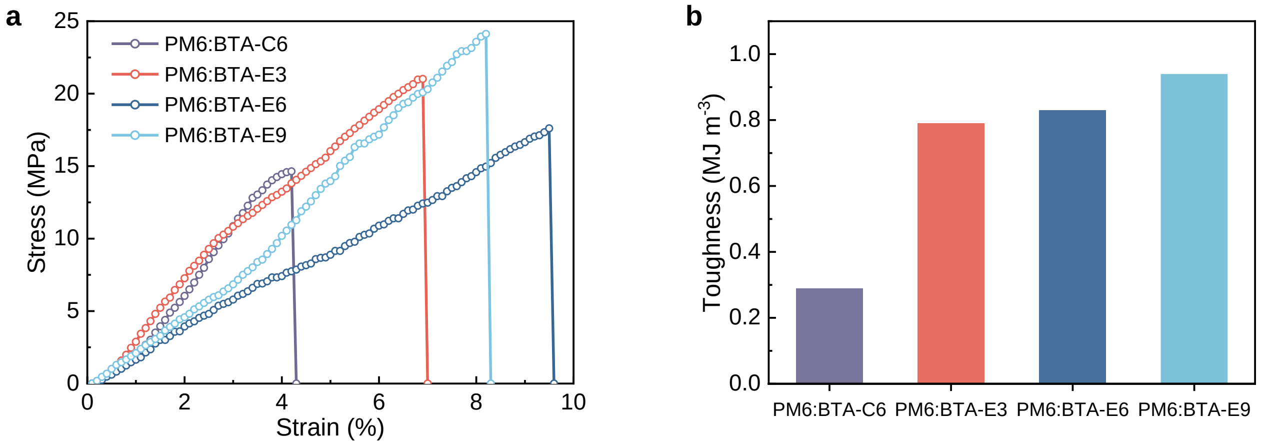

Supplementary Fig. 52 | Mechanical properties. **a,** Stress-strain curves of the PM6:BTA-C6, PM6:BTA-E3, PM6:BTA-E6 and PM6:BTA-E9 blend films by film-on-water measurement. **b,** Toughness of the PM6:BTA-C6, PM6:BTA-E3, PM6:BTA-E6 and PM6:BTA-E9 blend films. Source data are provided as a Source Data file.

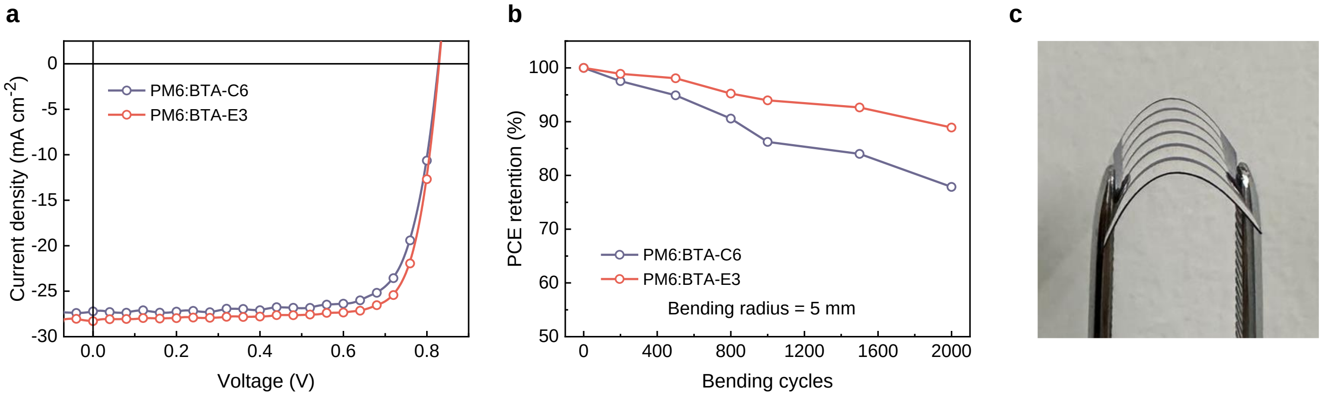

Supplementary Fig. 53 | Photovoltaic performance and mechanical durability of flexible OSCs. **a,** *J*-*V* curves of the flexible OSCs under the illumination of AM 1.5 G, 100 mW cm^{-2}. **b,** PCE retention of the flexible devices as a function of cycle number during the bending cyclic test. **c,** Photograph of the flexible OSC. Source data are provided as a Source Data file.

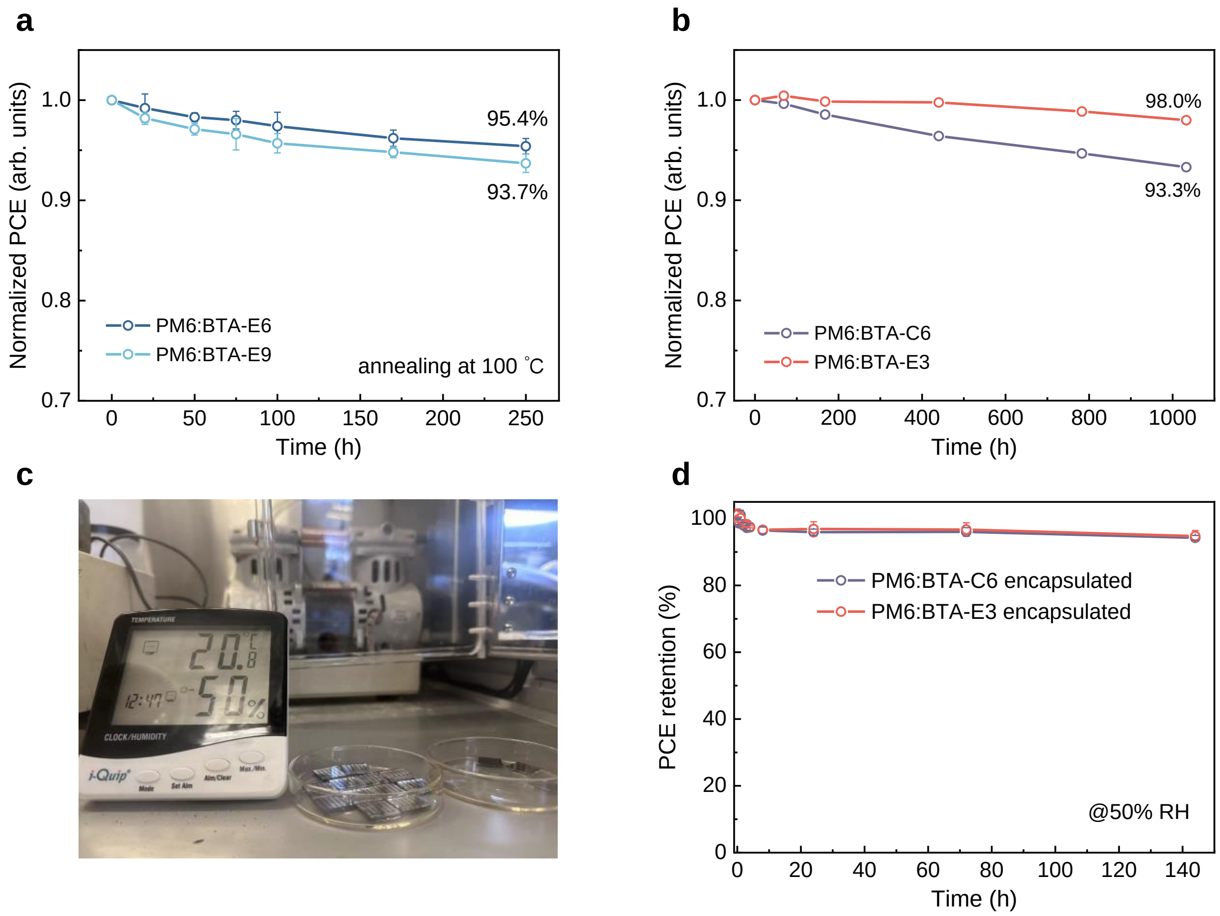

Supplementary Fig. 54 | Stability of OSCs. **a,** Normalized PCE of the OSCs based on PM6:BTA-E6 and PM6:BTA-E9 during annealing at 100 °C for 250 h. Error bars represent the standard error of the mean (n = 10). **b,** Normalized PCE of the PM6:BTA-C6 and PM6:BTA-E3 based OSCs under different aging time at the room temperature in nitrogen glove box. **c,** Photo of the stability test under 50% relative humidity condition. **d,** PCE retention of the encapsulated devices of the PM6:BTA-C6 and PM6:BTA-E3 during exposing to 50% relative humidity condition for 144 hours. Error bars represent the standard error of the mean (n = 5). Source data are provided as a Source Data file.

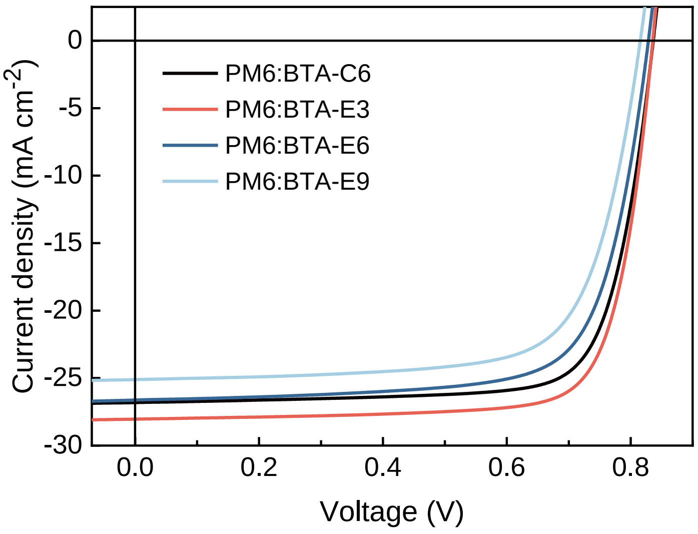

Supplementary Fig. 55 | *J-V* curves of the CF processed OSCs under the illumination of AM 1.5 G, 100 mW cm^{-2}.

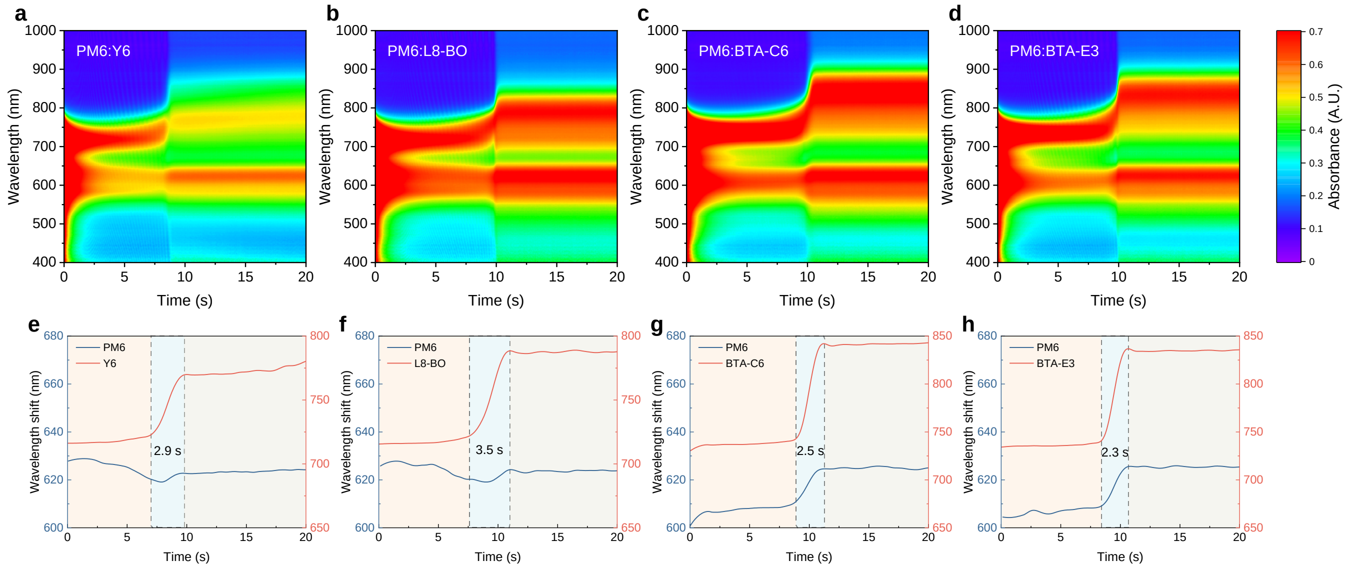

Supplementary Fig. 56 | Results of *in situ* UV-vis absorption. **a-d,** 2D *in situ* UV-vis absorption of PM6:Y6 (**a**), PM6:L8-BO (**b**), PM6:BTA-C6 (**c**) and PM6:BTA-E3 (**d**) blend films during film evolution. **e-h,** Time-dependent absorption peak shifts during film formation for the corresponding acceptors and PM6.

Supplementary Tables

Supplementary Table 1 | Summary of photovoltaic parameters of the recent non-halogenated solvent processed OSCs.

Active Layer	Solvent	V_{oc} (V)	J_{sc} (mA cm^{-2})	FF (%)	PCE (%)	Ref.
PM6:Y6:Y-4C-4O	*o*-XY	0.87	25.42	77.0	17.03	1
PM6:PTQ10:BTP-eC9	*o*-XY	0.855	27.86	80.2	19.10	2
D18:L8-BO	CS_2/PX	0.885	26.25	75.30	17.50	3
PM6:CH8-4	*o*-XY	0.900	25.51	75.1	17.27	4
PM6:G-Trimer	*o*-XY	0.896	26.75	79.30	19.01	5
PBQ6:PYF-T-*o*	Tol	0.886	25.12	76.64	17.06	6
PM6:BTP-eC9	Tol	0.865	27.43	76.55	18.16	7
PM6:Y6:BTO:PC_{71}BM	PX	0.85	27.12	75.75	17.41	8
D18-Cl:L8-BO-X	Tol	0.893	26.78	79.6	19.04	9
PM6: BTP-eC9	*o*-XY	0.846	28.2	78.8	18.8	10
PM6:CB16	*o*-XY	0.92	25.98	76.89	18.32	11
PM6:BTP-eC9	*o*-XY	0.851	27.65	79.0	18.6	12
PM6:BTP-eC9:PY-IT	*o*-XY	0.859	27.79	81.3	19.41	13
PM6:BTP-BO-4F:GS-ISO	*o*-XY	0.865	27.55	78.19	18.63	14
PM6:L8-BO:BTO-BO	Tol	0.881	27.14	81.23	19.42	15
PM6:D18-Cl:L8-BO	Tol/CS_2	0.872	27.29	79.10	18.83	16
PM6:L8-BO-X:Tri-V	*o*-XY	0.890	27.56	80.8	19.82	17
PDTP-BDD:D18:L8-BO	*o*-XY	0.905	26.90	79.53	19.36	18
D18:L8-BO:PY-TPT	*o*-XY	0.870	25.39	78.96	17.45	19
PL1:BTP-eC9-4F	*o*-XY	0.876	27.11	76.41	18.14	20
PM6:BTP-eC11:BN-T	*o*-XY	0.856	26.54	79.3	18.02	21
PM6:EV-i	*o*-XY	0.897	26.60	76.56	18.27	22
PM6:L8-Ph	*o*-XY	0.870	26.40	80.11	18.40	23
PM6:BTP-eC9	*o*-XY	0.847	27.22	80.31	18.52	24
D18:DTC11	*o*-XY/CS_2	0.858	27.5	80.5	19.0	25
PM6:L15	*o*-XY	0.93	25.95	77.26	18.72	26

Supplementary Table 2 | Energy loss of the devices based on PM6:BTA-E3, PM6:BTA-E6 and PM6:BTA-E9.

Devices	EQE_{EL}	E_g^{PV} (eV)	qV_{oc}^{SQ} (eV)	qV_{oc}^{rad} (eV)	qV_{oc}^{cal} (eV)	ΔE_1 (eV)	ΔE_2 (eV)	ΔE_3 (eV)	E_{loss} (eV)
PM6:BTA-E3	5.00×10^{-4}	1.340	1.083	1.037	0.840	0.257	0.046	0.197	0.500
PM6:BTA-E6	2.49×10^{-4}	1.339	1.081	1.037	0.823	0.258	0.044	0.214	0.516
PM6:BTA-E9	1.30×10^{-4}	1.339	1.081	1.032	0.801	0.258	0.049	0.231	0.538

Supplementary Table 3 | Charge dissociation and collection data of PM6:SMAs devices.

Active Layer	J_{ph} (mA cm^{-2})	J_{sat} (mA cm^{-2})	P_{diss}
PM6:BTA-C6	26.78	27.79	96.39%
PM6:BTA-E3	27.61	28.42	97.15%
PM6:BTA-E6	26.55	27.43	96.77%
PM6:BTA-E9	25.26	26.30	96.06%

Supplementary Table 4 | Hole and electron mobility data of PM6:SMAs devices.

Active Layer	μ_h^a (cm^2 V^{-1} s^{-1})	μ_e^a (cm^2 V^{-1} s^{-1})	μ_e/μ_h
PM6:BTA-C6	$(4.30 \pm 0.15)\times10^{-4}$	$(4.84 \pm 0.20)\times10^{-4}$	1.13
PM6:BTA-E3	$(3.08 \pm 0.18)\times10^{-4}$	$(4.54 \pm 0.19)\times10^{-4}$	1.47
PM6:BTA-E6	$(3.45 \pm 0.17)\times10^{-4}$	$(4.64 \pm 0.20)\times10^{-4}$	1.35
PM6:BTA-E9	$(4.93 \pm 0.15)\times10^{-4}$	$(5.18 \pm 0.16)\times10^{-4}$	1.05

[a] Average from 5 devices.

Supplementary Table 5 | GIWAXS data of all the films.

Samples	Direction	q (Å^{-1})	d-spacing (Å)	FWHM (Å^{-1})	CCL (Å)
PM6	IP	0.283	22.21	0.129	43.95
	OOP	1.70	3.69	0.370	15.29
BTA-C6	IP	0.422	14.88	0.117	48.19
	OOP	1.76	3.58	0.325	17.42
BTA-E3	IP	0.424	14.81	0.115	49.22
	OOP	1.76	3.58	0.302	18.72
BTA-E6	IP	0.422	14.89	0.132	42.92
	OOP	1.76	3.58	0.434	13.03
BTA-E9	IP	0.420	14.96	0.136	41.49
	OOP	1.76	3.58	0.433	13.06
PM6:BTA-C6	IP	0.302	20.79	0.074	76.86
		0.416	15.12	0.092	61.43
	OOP	1.78	3.53	0.287	19.67
PM6:BTA-E3	IP	0.303	20.73	0.073	77.03
		0.413	15.21	0.095	59.33
	OOP	1.79	3.51	0.272	20.78
PM6:BTA-E6	IP	0.297	21.15	0.065	86.36
		0.433	14.50	0.042	133.37
	OOP	1.75	3.60	---	---
PM6:BTA-E9	IP	0.296	21.26	0.056	101.72
		0.431	14.59	0.038	150.32
	OOP	1.75	3.59	---	---

Supplementary Table 6 | Photovoltaic parameters of the flexible OSCs under the illumination of AM 1.5 G, 100 mW cm^{-2}.

Active Layer	V_{oc} (V)	J_{sc} (mA cm^{-2})	FF (%)	PCE (%)	PCE retention[a] (%)
PM6:BTA-C6	0.828	27.28	76.02	17.18	77.85
PM6:BTA-E3	0.829	28.18	78.38	18.33	88.91

[a] The PCE retention after 2000 bending circles.

Supplementary Table 7 | Photovoltaic parameters of the CF processed OSCs under the illumination of AM 1.5 G, 100 mW cm^{-2}.

Active Layer	V_{oc} (V)	J_{sc} (mA cm^{-2})	FF (%)	PCE (%)
PM6:BTA-C6	0.837	26.82	76.73	17.22
PM6:BTA-E3	0.836	28.05	77.79	18.24
PM6:BTA-E6	0.829	26.64	73.01	16.13
PM6:BTA-E9	0.816	25.12	71.80	14.72

Supplementary Methods

The synthetic routes of BTA-E3, BTA-E6 and BTA-E9 were shown in Supplementary Figs. 1, 2, and 3 respectively. The detailed synthesis processes were described in the following.

Synthesis of compound E3-1

A mixture of compound BTA-2Br (3.00 g, 10.9 mmol), ethyl 3-bromopropanoate (2.35 g, 13.1 mmol) and anhydrous K_2CO_3 (1.81 g, 13.1 mmol) were added into a 100 mL flask and dissolved in 10 mL anhydrous DMF. The mixture was stirred at 90°C overnight under N_2 protection. After cooling down to room temperature, the mixture was poured into 300 mL water and extracted three times with dichloromethane. The obtained organic phase was dried over anhydrous $MgSO_4$ and then the solvent was removed by rotary evaporation. The crude product was purified by silica gel column chromatography with dichloromethane as the eluent to obtain compound **E3-1** as a white solid (1.43 g, 35.0% yield). ^{1}H NMR (300 MHz, Chloroform-*d*) δ 7.45 (s, 2H), 5.09 (t, *J* = 7.2 Hz, 2H), 4.20 (q, *J* = 7.1 Hz, 2H), 3.21 (t, *J* = 7.2 Hz, 2H), 1.26 (t, *J* = 7.1 Hz, 3H). ^{13}C NMR (101 MHz, $CDCl_3$) δ 169.82, 143.82, 129.81, 110.07, 61.23, 52.56, 34.17, 14.16.

Synthesis of compound E3-2.

Fuming nitric acid (0.84 g, 13.35 mmol) was added dropwise to fuming trifluoromethanesulfonic acid (8.01 g, 53.40 mmol) in a 100 mL flask at 0 °C. A white insoluble mixture was produced immediately, and then compound **E3-1** (1.00 g, 2.67 mmol) was added in portions over 20 min. After stirring at 80°C overnight, the mixture was poured into ice water slowly and neutralized with Na_2SO_3 and then extracted three times with dichloromethane. The obtained organic phase was dried over anhydrous $MgSO_4$ and then the solvent was removed by rotary evaporation. The crude product was purified by silica gel column chromatography with dichloromethane as the eluent to obtain compound **E3-2** as a white solid (0.96 g, 77.3% yield). ^{1}H NMR (400 MHz, Chloroform-*d*) δ 5.16 (t, *J* = 6.8 Hz, 2H), 4.19 (q, *J* = 7.1 Hz, 2H), 3.26 (t, *J* = 6.8 Hz, 2H), 1.26 (t, *J* = 7.1 Hz, 3H). ^{13}C NMR (101 MHz, $CDCl_3$) δ 169.33, 143.25, 142.00, 107.37, 61.52, 53.81, 33.52, 14.15.

Synthesis of compound E3-3.

A mixture of compound **E3-2** (0.96 g, 2.07 mmol), tributyl(6-hexylthieno[3,2-*b*]thiophen-2-yl)stannane (3.19 g, 6.20 mmol) and $Pd(PPh_3)_4$ (115 mg, 0.10 mmol) were added into a 100 mL flask and dissolved in 10 mL anhydrous toluene. The mixture was stirred at 110°C overnight under N_2 protection. After cooling down to room temperature, the mixture was poured into 300 mL water and extracted three times with dichloromethane. The obtained organic phase was dried over anhydrous $MgSO_4$ and then the solvent was removed by rotary evaporation. The crude product was purified by silica gel column chromatography with dichloromethane/petroleum ether (1/1, *v*/*v*) as the eluent to obtain compound **E3-3** as a red solid (1.03 g, 66.1% yield). ^{1}H NMR (400 MHz, Chloroform-d) δ 7.73 (s, 2H), 7.13 (s, 2H), 5.15 (t, J = 7.0 Hz, 2H), 4.18 (q, J = 7.3 Hz, 2H), 3.22 (t, J = 6.9 Hz, 2H), 2.75 (t, J = 7.7 Hz, 4H), 1.77 (p, J = 7.5 Hz, 4H), 1.45-1.38 (m, 4H), 1.34 (m,

8H), 1.22 (t, J = 7.2 Hz, 3H), 0.94-0.87 (m, 6H). ^{13}C NMR (101 MHz, $CDCl_3$) δ 169.64, 143.53, 141.74, 139.85, 139.05, 135.07, 130.13, 124.78, 123.76, 119.74, 61.41, 53.27, 33.77, 31.61, 29.84, 29.08, 28.57, 22.64, 14.15, 14.13. MS (MALDI-TOF) *m*/*z*: $[M+H]^+$ calcd for $C_{35}H_{39}N_5O_6S_4$, 754.18, found: 754.19.

Synthesis of compound E3-4.

A mixture of compound **E3-3** (1.03 g, 1.37 mmol) and PPh_3 (3.59 g, 13.70 mmol) were added into a 100 mL flask and dissolved in 10 mL NMP. The mixture was stirred at 180°C overnight under N_2 protection. After cooling down to room temperature, 5-(bromomethyl)undecane (1.70 g, 6.85 mmol), KI (0.23 g, 1.37 mmol) and K_2CO_3 (1.89 g, 13.70 mmol) were directedly added into the flask and the mixture was deoxygenated with N_2 for 5 min. The mixture was stirred at 100°C for 15 h. After cooling down to room temperature, the mixture was poured into 500 mL water and extracted three times with dichloromethane. The obtained organic phase was dried over anhydrous $MgSO_4$ and then the solvent was removed by rotary evaporation. The crude product was purified by silica gel column chromatography with dichloromethane/petroleum ether (1/1, *v*/*v*) as the eluent to obtain compound **E3-4** as a yellow solid (503 mg, 34.4% yield). ^{1}H NMR (400 MHz, Chloroform-*d*) δ 6.98 (s, 2H), 5.14 (t, *J* = 7.4 Hz, 2H), 4.57 (d, *J* = 7.7 Hz, 4H), 4.25 (q, *J* = 7.1 Hz, 2H), 3.26 (t, *J* = 7.4 Hz, 2H), 2.81 (t, *J* = 7.8 Hz, 4H), 2.01 (p, *J* = 6.6 Hz, 2H), 1.85 (p, *J* = 7.6 Hz, 4H), 1.47-1.14 (m, 23H), 1.11- 0.74 (m, 40H), 0.71-0.50 (m, 16H). ^{13}C NMR (101 MHz, $CDCl_3$) δ 170.71, 141.52, 137.13, 136.71, 136.25, 130.95, 123.97, 121.29, 118.77, 109.16, 60.94, 54.81, 51.28, 38.57, 34.82, 31.68, 31.56, 30.24, 29.68, 29.33, 29.18, 28.82, 27.79, 25.28, 22.69, 22.63, 22.44, 14.21, 14.10, 13.94, 13.72, 13.68. MS (MALDI-TOF) *m*/*z*: $[M+H]^+$ calcd for $C_{59}H_{87}N_5O_2S_4$, 1025.57, found: 1025.57.

Synthesis of compound E3-5.

To a solution of compound **E3-4** (503 mg, 0.47 mmol) and DMF (2.3 mL) in $CHCl_3$ (10 mL) at 0°C, $POCl_3$ (1.1 mL) was added dropwise slowly under N_2 protection. After stirring at the room temperature for 1 h, the mixture was stirred at 65°C overnight. After cooling down to room temperature, saturated sodium acetate aqueous solution was added into the flask and the mixture was stirred for an additional 1 h. The mixture was poured into 300 mL water and extracted three times with dichloromethane. The obtained organic phase was dried over anhydrous $MgSO_4$ and then the solvent was removed by rotary evaporation. The crude product was purified by silica gel column chromatography with dichloromethane/petroleum ether (4/1, *v*/*v*) as the eluent to obtain compound **E3-5** (457 mg, 86.5% yield) as a yellow solid. ^{1}H NMR (400 MHz, Chloroform-*d*) δ 10.13 (s, 2H), 5.14 (t, *J* = 7.3 Hz, 2H), 4.60 (d, *J* = 7.8 Hz, 4H), 4.25 (q, *J* = 7.1 Hz, 2H), 3.27 (t, *J* = 7.3 Hz, 2H), 3.19 (t, *J* = 7.7 Hz, 4H), 2.03-1.86 (m, 6H), 1.7-1.55 (m, 2H), 1.54-1.17 (m, 28H), 1.10-0.75 (m, 47H), 0.75-0.41 (m, 17H). ^{13}C NMR (101 MHz, $CDCl_3$) δ 181.62, 170.48, 146.73, 142.74, 136.92, 136.70, 136.15, 132.32, 130.01, 126.12, 110.29, 61.01, 55.07, 51.46, 38.80, 35.42, 34.63, 31.54, 30.31, 29.29, 28.16, 27.88, 26.91, 26.43, 24.98, 22.69, 22.52, 22.40, 14.20, 14.02, 13.90, 13.67, 13.63. MS (MALDI-TOF) *m*/*z*: $[M+H]^+$ calcd for $C_{61}H_{87}N_5O_4S_4$, 1081.56, found: 1081.56.

Synthesis of BTA-E3.

A mixture of compound **E3-5** (457 mg, 0.41 mmol), pyridine (1.0 mL) and 2-(5,6-difluoro-3-oxo-2,3-dihydro-1*H*-inden-1-ylidene)malononitrile (377 mg, 1.64 mmol) were added into a 100 mL flask and dissolved in 10 mL $CHCl_3$. The mixture was stirred at room temperature overnight under N_2 protection. Then the mixture was poured into methanol and filtered. The crude product was purified by silica gel column chromatography with dichloromethane/petroleum ether (4/1, *v*/*v*) as the eluent to obtain **BTZ-E3** as a dark blue solid (439 mg, 71.1% yield). ^{1}H NMR (400 MHz, Chloroform-*d*) δ 9.16 (s, 2H), 8.56 (dd, *J* = 10.0, 6.4 Hz, 2H), 7.68 (t, *J* = 7.5 Hz, 2H), 5.15 (t, *J* = 7.2 Hz, 2H), 4.73 (d, *J* = 7.8 Hz, 4H), 4.26 (q, *J* = 7.1 Hz, 2H), 3.32-3.16 (m, 6H), 2.05 (p, *J* = 6.7 Hz, 2H), 1.88 (p, *J* = 7.8 Hz, 4H), 1.56-1.50 (m, 4H) 1.42-1.27 (m, 12H), 1.22-0.82 (m, 36H), 0.72-0.59 (m, 15H). ^{13}C NMR (101 MHz, $CDCl_3$) δ 186.06, 170.42, 155.66, 155.53, 153.09, 153.04, 152.96, 152.90, 136.65, 136.61, 136.18, 134.52, 134.48, 133.83, 129.88, 115.07, 115.00, 114.79, 114.70, 112.43, 112.25, 61.14, 55.47, 51.59, 39.11, 34.49, 31.63, 31.20, 30.47, 30.31, 29.87, 29.52, 29.38, 28.05, 25.16, 22.83, 22.54, 22.47, 14.24, 14.08, 14.01, 13.79, 13.75. MS (MALDI-TOF) *m*/*z*: $[M+H]^+$ calcd for $C_{85}H_{91}F_4N_9O_4S_4$, 1505.60, found: 1505.60.

Synthesis of compound E6-2.

A mixture of compound **E6-1** (1.98 g, 2.49 mmol) and PPh_3 (6.53 g, 24.9 mmol) were added into a 100 mL flask and dissolved in 10 mL NMP. The mixture was stirred at 180°C overnight under N_2 protection. After cooling down to room temperature, 5-(bromomethyl)undecane (3.09 g, 12.45 mmol), KI (0.41 g, 2.49 mmol) and K_2CO_3 (3.44 g, 24.9 mmol) were directedly added into the flask and the mixture was deoxygenated with N_2 for 5 min. The mixture was stirred at 100°C for 15 h. After cooling down to room temperature, the mixture was poured into 500 mL water and extracted three times with dichloromethane. The obtained organic phase was dried over anhydrous $MgSO_4$ and then the solvent was removed by rotary evaporation. The crude product was purified by silica gel column chromatography with dichloromethane/petroleum ether (2/1, *v*/*v*) as the eluent to obtain compound **E6-2** as a yellow solid (937 mg, 35.3% yield). ^{1}H NMR (300 MHz, Chloroform-*d*) δ 6.98 (s, 2H), 4.84 (t, *J* = 7.1 Hz, 2H), 4.57 (d, *J* = 7.7 Hz, 4H), 4.13 (q, *J* = 6.8 Hz, 2H), 2.81 (t, *J* = 7.6 Hz, 4H), 2.43 (t, *J* = 7.4 Hz, 2H), 2.38-2.19 (m, 2H), 2.00 (p, *J* = 7.7, 6.9 Hz, 2H), 1.91-1.71 (m, 6H), 1.60-1.14 (m, 43H), 1.11-0.72 (m, 52H), 0.72-0.51 (m, 19H). MS (MALDI-TOF) m/z: $[M+H]^+$ calcd for $C_{62}H_{93}N_5O_2S_4$, 1067.62, found: 1067.62.

Synthesis of compound E6-3.

To a solution of compound **E6-2** (937 mg, 0.88 mmol) and DMF (4.1 mL) in $CHCl_3$ (10 mL) at 0°C, $POCl_3$ (2.0 mL) was added dropwise slowly under N_2 protection. After stirring at the room temperature for 1 h, the mixture was stirred at 65°C overnight. After cooling down to room temperature, saturated sodium acetate aqueous solution was added into the flask and the mixture was stirred for an additional 1 h. The mixture was poured into 300 mL water and extracted three times with dichloromethane. The obtained organic phase was dried over anhydrous $MgSO_4$ and then the solvent was removed by rotary

evaporation. The crude product was purified by silica gel column chromatography with dichloromethane/petroleum ether (4/1, *v*/*v*) as the eluent to obtain compound **E6-3** (896 mg, 90.6% yield) as a yellow solid. ^{1}H NMR (400 MHz, Chloroform-*d*) δ 10.13 (s, 2H), 4.85 (t, *J* = 7.6 Hz, 2H), 4.60 (d, *J* = 7.8 Hz, 4H), 4.15 (q, *J* = 6.4, 5.7 Hz, 2H), 3.20 (t, *J* = 7.8 Hz, 4H), 2.44 (t, *J* = 7.4 Hz, 2H), 2.30-2.23 (m, 2H), 1.98-1.88 (m, 6H), 1.84-1.76 (m, 4H), 1.53-1.43 (m, 6H), 1.36-1.24 (m, 22H), 1.00-0.84 (m, 29H), 0.70-0.55 (m, 17H). ^{13}C NMR (101 MHz, $CDCl_3$) δ 181.69, 173.16, 146.83, 142.70, 136.89, 136.64, 135.99, 132.28, 130.06, 126.13, 110.38, 60.31, 55.74, 55.00, 38.80, 37.11, 34.12, 33.72, 31.57, 30.34, 30.12, 29.71, 29.33, 28.19, 27.89, 26.25, 25.15, 24.52, 22.72, 22.56, 22.43, 14.26, 14.14, 14.08, 13.95, 13.72. MS (MALDI-TOF) m/z: $[M+H]^+$ calcd for $C_{64}H_{93}N_5O_4S_4$, 1123.61, found: 1123.61.

Synthesis of BTA-E6.

A mixture of compound **E6-3** (896 mg, 0.80 mmol), pyridine (2.0 mL) and 2-(5,6-difluoro-3-oxo-2,3-dihydro-1*H*-inden-1-ylidene)malononitrile (736 mg, 3.20 mmol) were added into a 100 mL flask and dissolved in 10 mL $CHCl_3$. The mixture was stirred at room temperature overnight under N_2 protection. Then the mixture was poured into methanol and filtered. The crude product was purified by silica gel column chromatography with dichloromethane/petroleum ether (4/1, *v*/*v*) as the eluent to obtain **BTZ-E6** as a dark blue solid (857 mg, 69.2% yield). ^{1}H NMR (400 MHz, Chloroform-*d*) δ 9.14 (s, 2H), 8.55 (dd, *J* = 10.0, 6.4 Hz, 2H), 7.68 (t, *J* = 7.5 Hz, 2H), 4.86 (t, *J* = 7.7 Hz, 2H), 4.75 (d, *J* = 7.7 Hz, 4H), 4.16 (q, *J* = 7.1 Hz, 2H), 3.22 (t, *J* = 7.9 Hz, 4H), 2.45 (t, *J* = 7.4 Hz, 2H), 2.33-2.22 (m, 2H), 2.06 (p, *J* = 6.6 Hz, 2H), 1.94-1.74 (m, 6H), 1.52 (dt, *J* = 14.4, 7.3 Hz, 5H), 1.38-0.81 (m, 48H), 0.72-0.56 (m, 14H). ^{13}C NMR (101 MHz, $CDCl_3$) δ 186.05, 173.44, 173.11, 155.65, 155.51, 153.08, 153.02, 152.94, 152.89, 136.66, 136.60, 135.98, 134.53, 134.47, 133.77, 129.94, 115.11, 114.99, 114.77, 114.72, 112.42, 112.24, 60.46, 56.15, 55.91, 55.46, 39.12, 34.10, 33.68, 31.64, 31.23, 30.48, 29.88, 29.53, 28.06, 27.84, 26.23, 25.38, 25.15, 24.49, 22.83, 22.54, 22.11, 14.27, 14.08, 14.01, 13.78, 13.74. MS (MALDI-TOF) *m*/*z*: $[M+H]^+$ calcd for $C_{88}H_{97}F_4N_9O_4S_4$, 1547.65, found: 1547.65.

Synthesis of compound E9-2.

A mixture of compound **E9-1** (1.42 g, 1.70 mmol) and PPh_3 (4.46 g, 17.0 mmol) were added into a 100 mL flask and dissolved in 10 mL NMP. The mixture was stirred at 180°C overnight under N_2 protection. After cooling down to room temperature, 5-(bromomethyl)undecane (2.11 g, 8.50 mmol), KI (0.28 g, 1.70 mmol) and K_2CO_3 (2.35 g, 17.0 mmol) were directedly added into the flask and the mixture was deoxygenated with N_2 for 5 min. The mixture was stirred at 100°C for 15 h. After cooling down to room temperature, the mixture was poured into 500 mL water and extracted three times with dichloromethane. The obtained organic phase was dried over anhydrous $MgSO_4$ and then the solvent was removed by rotary evaporation. The crude product was purified by silica gel column chromatography with dichloromethane/petroleum ether (2/1, *v*/*v*) as the eluent to obtain compound **E9-2** as a yellow solid (798 mg, 42.3% yield). ^{1}H NMR (300 MHz, Chloroform-*d*) δ 6.98 (s, 2H), 4.81 (t, *J* = 7.5 Hz, 2H), 4.57 (d, *J* = 7.7 Hz, 4H), 4.12 (q, *J* = 7.1 Hz, 2H), 2.81 (t, *J* = 7.7 Hz, 4H), 2.28 (t, *J* = 7.5 Hz, 2H),

2.25-2.15 (m, 2H), 2.07-1.95 (m, 2H), 1.85 (p, *J* = 7.4 Hz, 2H), 1.66-1.56 (m, 2H), 1.49-1.20 (m, 29H), 0.99-0.75 (m, 34H), 0.72-0.51 (m, 16H). MS (MALDI-TOF) m/z: $[M+H]^+$ calcd for $C_{65}H_{99}N_5O_2S_4$, 1109.67, found: 1109.67.

Synthesis of compound E9-3.

To a solution of compound **E9-2** (798 mg, 0.72 mmol) and DMF (3.3 mL) in $CHCl_3$ (10 mL) at 0°C, $POCl_3$ (1.6 mL) was added dropwise slowly under N_2 protection. After stirring at the room temperature for 1 h, the mixture was stirred at 65°C overnight. After cooling down to room temperature, saturated sodium acetate aqueous solution was added into the flask and the mixture was stirred for an additional 1 h. The mixture was poured into 300 mL water and extracted three times with dichloromethane. The obtained organic phase was dried over anhydrous $MgSO_4$ and then the solvent was removed by rotary evaporation. The crude product was purified by silica gel column chromatography with dichloromethane/petroleum ether (4/1, *v*/*v*) as the eluent to obtain compound **E9-3** (767 mg, 91.4% yield) as a yellow solid. ^{1}H NMR (400 MHz, Chloroform-*d*) δ 10.13 (s, 2H), 4.82 (t, *J* = 7.4 Hz, 2H), 4.60 (d, *J* = 7.8 Hz, 4H), 4.12 (q, *J* = 7.1 Hz, 2H), 3.20 (t, *J* = 7.7 Hz, 4H), 2.28 (t, *J* = 7.4 Hz, 2H), 2.25-2.22 (m, 2H), 1.98-1.88 (m, 6H), 1.66-1.59 m, 3H), 1.49-1.43 (m, 9H), 1.39-1.15 (m, 52H), 1.02-0.76 (m, 51H), 0.74-0.48 (m, 17H). ^{13}C NMR (101 MHz, $CDCl_3$) δ 181.67, 173.81, 146.82, 142.71, 136.91, 136.63, 135.93, 132.26, 130.08, 126.16, 110.46, 60.17, 56.27, 55.05, 38.82, 37.12, 34.35, 32.77, 31.94, 31.57, 30.35, 29.71, 29.33, 29.07, 28.20, 27.91, 27.72, 27.10, 26.67, 24.94, 22.72, 22.56, 22.43, 19.74, 14.27, 14.13, 14.06, 13.94, 13.70. MS (MALDI-TOF) m/z: $[M+H]^+$ calcd for $C_{67}H_{99}N_5O_4S_4$, 1165.66, found: 1165.66.

Synthesis of BTA-E9.

A mixture of compound **E9-3** (767 mg, 0.66 mmol), pyridine (1.6 mL) and 2-(5,6-difluoro-3-oxo-2,3-dihydro-1*H*-inden-1-ylidene)malononitrile (607 mg, 2.64 mmol) were added into a 100 mL flask and dissolved in 10 mL $CHCl_3$. The mixture was stirred at room temperature overnight under N_2 protection. Then the mixture was poured into methanol and filtered. The crude product was purified by silica gel column chromatography with dichloromethane/petroleum ether (4/1, *v*/*v*) as the eluent to obtain **BTZ-E9** as a dark blue solid (712 mg，67.9% yield). ^{1}H NMR (400 MHz, Chloroform-*d*) δ 9.17 (s, 2H), 8.57 (dd, *J* = 10.0, 6.4 Hz, 2H), 7.69 (t, *J* = 7.4 Hz, 2H), 4.82 (t, *J* = 7.5 Hz, 2H), 4.73 (d, *J* = 7.8 Hz, 4H), 4.12 (q, *J* = 7.1 Hz, 2H), 3.23 (s, 4H), 2.29 (t, *J* = 7.4 Hz, 2H), 2.22 (p, *J* = 7.2 Hz, 2H), 2.04 (p, *J* = 6.5 Hz, 2H), 1.88 (p, *J* = 7.8 Hz, 4H), 1.63 (p, *J* = 7.8 Hz, 2H), 1.57-1.22 (m, 26H), 1.19-0.78 (m, 37H), 0.74-0.55 (m, 14H). ^{13}C NMR (101 MHz, $CDCl_3$) δ 180.64, 173.80, 155.76, 155.64, 155.52, 153.03, 152.97, 152.90, 136.71, 136.61, 136.36, 134.54, 134.46, 133.54, 129.96, 115.01, 114.78, 114.62, 112.43, 112.25, 111.86, 60.20, 56.43, 55.44, 39.10, 34.33, 31.64, 31.58, 30.44, 30.30, 29.54, 29.36, 29.06, 28.97, 28.06, 27.83, 26.65, 25.35, 25.11, 24.92, 22.82, 22.76, 22.54, 22.46, 14.27, 14.08, 14.03, 13.78, 13.73. MS (MALDI-TOF) m/z: $[M+H]^+$ calcd for $C_{91}H_{103}F_4N_9O_4S_4$, 1589.70, found: 1589.69.

Supplementary Notes

Supplementary Note I

The CF processed devices was fabricated with the conventional structure of ITO/PEDOT:PSS/ active layers/PDINN/Ag. The *J-V* curves of the optimized CF processed OSCs are shown in Supplementary Fig. 55, and the corresponding photovoltaic performance parameters of the CF processed devices are summarized in Supplementary Table 7. When processed with CF, all the four acceptors exhibited almost identical PCE with that of *o*-XY processed devices, with the corresponding PCE of 17.22%, 18.24%, 16.13% and 14.72% for the PM6:BTA-C6, PM6:BTA-E3, PM6:BTA-E6 and PM6:BTA-E9 respectively. Taking into account environmental concerns, we have opted for the eco-friendly green solvent *o*-XY as the processing solvent.

Supplementary Note II

To elucidate the evolution of the blend film during the coating process, the *in situ* UV-vis absorption spectra of the blend films processed with *o*-xylene were monitored. The widely used and represent acceptors Y6 and L8-BO were chosen for comparison with the BTA-C6 and BTA-E3 to investigate the role of H-bonding in non-halogenated solvent processing. As depicted in Supplementary Figs. 56a-d, the film formation process could be divided into three stages. In stage I, the blend solutions ware spin-coated on the substate and the absorption peak location of acceptors remained almost unchanged. In stage II, due to the solvent evaporation, the solution concentration exceeded the solubility limit and caused a rapid red-shift in the absorption peak of acceptors. Finally, upon complete solvent evaporation, the blend film was ultimately formed and both the absorption peak location and intensity reached a constant value (stage III). Among the three stages, the stage II is identified as critical to film evolution as it determines the film morphology.

As shown in Supplementary Figs. 56e-f, the duration of stage II of the PM6:Y6 and PM6:L8-BO blend films was determined to be 2.9 s and 3.5 s. The long crystalline time may result in excessive aggregation of Y6 and L8-BO, leading to the unfavorable morphology in the *o*-xylene processed blend films. Moreover, the absorption peak locations of PM6 in the stage I of the PM6:Y6 and PM6:L8-BO solutions were found to be about 627 nm, indicating that the PM6 suffered severe entanglement in their solutions. In addition, the crystallization of PM6 in the PM6:Y6 and PM6:L8-BO solutions was determined significantly faster than of the acceptors. Consequently, the PM6:Y6 and PM6:L8-BO blend films could easily form the unfavorable phase separation and morphology. In terms of the PM6:BTA-C6 blend films (Supplementary Fig. 56g), the peak location of PM6 in the PM6:BTA-C6 blend film started to show the gradually redshift at 7.7 s, while the absorption peak of the BTA-C6 barely changed at the same time, which suggested that donor aggregation came before the acceptor during the film evolution. Additionally, the PM6 also exhibited longer assembly duration (from 7.7 s to 11.0 s) than that of BTA-C6 (from 8.8 s to 11.3 s), and the long assembly process might lead to the excessive aggregation and large

domain size as evidenced by the AFM and TEM imagine (Fig. 5). While for the PM6:BTA-E3 blend film (Supplementary Fig. 56h), the absorption peak of PM6 and BTA-E3 almost changed at the same time (from 8.4 s to 10.7 s), indicating that the donor and acceptor crystallized simultaneously. Besides, the PM6:BTA-E3 blend films exhibited the shorter film formation duration (c.a. 2.3 s) than that of PM6:BTA-C6 counterpart (c.a. 2.5 s), which was mainly resulted from the enhanced homogeneous and heterogeneous interactions. As a result, benefit from the introduction of H-bonding, the PM6:BTA-E3 blend film exhibited a rapid and synchronous film evolution process, resulting in optimal phase separation along with the fiber-like bicontinuous network (Fig. 5), which is conducive to charge generation and extraction and contributing to the improved device performance.

Supplementary References

1. Kim, C. *et al.* Impact of the molecular structure of oligo(ethylene glycol)-incorporated Y-series acceptors on the formation of alloy-like acceptors and performance of non-halogenated solvent-processable organic solar cells. *ACS Appl. Mater. Interfaces* **15**, 24670–24680 (2023).
2. Ma, R. *et al.* Revealing the underlying solvent effect on film morphology in high-efficiency organic solar cells through combined *ex situ* and *in situ* observations. *Energy Environ. Sci.* **16**, 2316–2326 (2023).
3. Su, Y. *et al.* High-efficiency organic solar cells processed from a halogen-free solvent system. *Sci. China Chem.* **66**, 2380–2388 (2023).
4. Chen, H. *et al.* Terminally chlorinated and thiophene-linked acceptor-donor-acceptor structured 3D acceptors with versatile processability for high-efficiency organic solar cells. *Angew. Chem. Int. Ed.* **62**, e202307962 (2023).
5. Wang, C. *et al.* Unique assembly of giant star-shaped trimer enables non-halogen solvent-fabricated, thermal stable, and efficient organic solar cells. *Joule* **7**, 2386–2401 (2023).
6. Hu, K. *et al.* Solid additive tuning of polymer blend morphology enables non-halogenated-solvent all-polymer solar cells with an efficiency of over 17%. *Energy Environ. Sci.* **15**, 4157–4166 (2022).
7. Zhang, J. *et al.* Polymer-entangled spontaneous pseudo planar heterojunction for constructing efficient flexible organic solar cells. *Adv. Mater.* **36**, 2309379 (2023).
8. Chen, H. *et al.* A guest-assisted molecular-organization approach for >17% efficiency organic solar cells using environmentally friendly solvents. *Nat. Energy* **6**, 1045–1053 (2021).
9. Luo, S. *et al.* Auxiliary sequential deposition enables 19%-efficiency organic solar cells processed from halogen-free solvents. *Nat. Commun.* **14**, 6964 (2023).
10. He, W. *et al.* In situ self-assembly of trichlorobenzoic acid enabling organic photovoltaics with approaching 19% efficiency. *Adv. Funct. Mater.* **34**, 2313594 (2023).
11. Xue, Y.-J. *et al.* Unraveling the structure–property–performance relationships of fused-ring nonfullerene acceptors: toward a C-shaped *ortho* -benzodipyrrole-based acceptor for highly efficient organic photovoltaics. *J. Am. Chem. Soc.* **146**, 833–848 (2023).
12. Xu, L. *et al.* Volatile solid-assisted molecular assembly enables eco-friendly processed organic photovoltaic cells with high efficiency and photostability. *Adv. Funct. Mater.* **34**, 2314178 (2024).
13. Zhang, Y. *et al.* Achieving 19.4% organic solar cell via an *in situ* formation of p-i-n structure with built-in interpenetrating network. *Joule* **8**, 509–526 (2024).
14. Huang, T. *et al.* 18.63% efficiency of ternary organic solar cells achieved via nonhalogenated solvent and hot spin-coating

process. *Adv. Funct. Mater.* **34**, 2315825 (2024).

15.Chen, H. *et al.* Heterogeneous nucleating agent for high-boiling-point nonhalogenated solvent-processed organic solar cells and modules. *Adv. Mater.* **36**, 2402350 (2024).

16.Zhang, Z. *et al.* Synchronous regulation of donor and acceptor microstructure using thiophene-derived non-halogenated solvent additives for efficient and stable organic solar cells. *Adv. Funct. Mater.* **34**, 2401823 (2024).

17.Song, J. *et al.* Non-halogenated solvent-processed organic solar cells with approaching 20 % efficiency and improved photostability. *Angew. Chem. Int. Ed.* **63**, e202404297 (2024).

18.He, Y. *et al.* Developing benzodithiophene-free donor polymer for 19.36% efficiency green-solvent-processable organic solar cells. *Chem. Eng. J.* **490**, 151920 (2024).

19.Wei, Y. *et al.* High performance as-cast organic solar cells enabled by a refined double-fibril network morphology and improved dielectric constant of active layer. *Adv. Mater.* **36**, 2403294 (2024).

20.Lu, H. *et al.* Random terpolymer enabling high-efficiency organic solar cells processed by nonhalogenated solvent with a low nonradiative energy loss. *Adv. Funct. Mater.* **32**, 2203193 (2022).

21.Ma, R. *et al. In situ* and *ex situ* investigations on ternary strategy and co-solvent effects towards high-efficiency organic solar cells. *Energy Environ. Sci.* **15**, 2479–2488 (2022).

22.Zhuo, H. *et al.* Giant molecule acceptor enables highly efficient organic solar cells processed using non-halogenated solvent. *Angew. Chem. Int. Ed.* **62**, e202303551 (2023).

23.Wu, X. *et al.* Introducing phenyl end group in the inner side chains of A-DA’D-A acceptors enables high-efficiency organic solar cells processed with nonhalogenated solvent. *Adv. Mater.* **35**, 2302946 (2023).

24.Yang, C. *et al.* Hot-casting strategy empowers high-boiling solvent-processed organic solar cells with over 18.5% efficiency. *Adv. Mater.* **36**, 2305356 (2024).

25.Zhong, Z. *et al.* Non-halogen solvent processed binary organic solar cells with efficiency of 19% and module efficiency over 15% enabled by asymmetric alkyl chain engineering. *Adv. Energy Mater.* **13**, 2302273 (2023).

26.Liu, B. *et al.* Isomerized green solid additive engineering for thermally stable and eco-friendly all-polymer solar cells with approaching 19% efficiency. *Adv. Mater.* **35**, 2308334 (2023).
