# Supplementary material for: Dynamic hydrogen-bonding enables high-performance and mechanically robust organic solar cells processed with non-halogenated solvent
Source: Nat Commun. 2025 Jan 17;16:787. doi: 10.1038/s41467-024-55375-8 (PMC11748654; doi:10.1038/s41467-024-55375-8)
Supplement: Supplementary file 3 — Reporting Summary [file 41467_2024_55375_MOESM3_ESM.pdf]

nature research

Corresponding author(s): Xiaojun Li, Yongfang Li

Solar Cells Reporting Summary

Nature Research wishes to improve the reproducibility of the work that we publish. This form is intended for publication with all accepted papers reporting the characterization of photovoltaic devices and provides structure for consistency and transparency in reporting. Some list items might not apply to an individual manuscript, but all fields must be completed for clarity.

For further information on Nature Research policies, including our data availability policy, see Authors & Referees.

Experimental design

Please check: are the following details reported in the manuscript?

1. Dimensions

Area of the tested solar cells
- [x] Yes
- [ ] No

Area of the tested solar cells is 0.1428 cm2 defined by optical microscope (Olympus BX51).

Method used to determine the device area
- [x] Yes
- [ ] No

Device area was determined by a mask of 0.09875 cm2. The area of the mask was certified by National Institute of Metrology (NIM, China).

2. Current-voltage characterization

Current density-voltage (J-V) plots in both forward and backward direction
- [ ] Yes
- [x] No

Generally, organic photovoltaic devices do not have forward and backward problems. And we only scan the device in forward direction.

Voltage scan conditions
For instance: scan direction, speed, dwell times
- [x] Yes
- [ ] No

The voltage was scanned from -0.2 V to 1.0 V. The voltage step and delay time were 20 mV and 1 ms, respectively. Relative information is provided in method section.

Test environment
For instance: characterization temperature, in air or in glove box
- [x] Yes
- [ ] No

Devices were characterized at room temperature in In N2-filled glove box.

Protocol for preconditioning of the device before its characterization
- [ ] Yes
- [x] No

No preconditioning protocol.

Stability of the J-V characteristic
Verified with time evolution of the maximum power point or with the photocurrent at maximum power point; see ref. 7 for details.
- [ ] Yes
- [x] No

Organic photovoltaic devices show no decay or instability during the test of J-V characteristics. The MPP output is consistent with the J-V curve.

3. Hysteresis or any other unusual behaviour

Description of the unusual behaviour observed during the characterization
- [ ] Yes
- [x] No

No hysteresis was observed in our device.

Related experimental data
- [ ] Yes
- [x] No

We didn't find the unusual behaviour.

4. Efficiency

External quantum efficiency (EQE) or incident photons to current efficiency (IPCE)
- [x] Yes
- [ ] No

The data is included in the manuscript. We show the EQE measurement details in the Methods section.

A comparison between the integrated response under the standard reference spectrum and the response measure under the simulator
- [x] Yes
- [ ] No

The difference between the integrated current from EQE and the short-circuit current from J-V curve measured under AM 1.5G solar simulator is within 5% difference which is within the accuracy confidence of the measurements.

For tandem solar cells, the bias illumination and bias voltage used for each subcell
- [ ] Yes
- [x] No

We did not make the tandem solar cells in this work.

5. Calibration

Light source and reference cell or sensor used for the characterization
- [x] Yes
- [ ] No

Relative information is provided in method section.

Confirmation that the reference cell was calibrated and certified
- [x] Yes
- [ ] No

Relative information is provided in method section.

Calculation of spectral mismatch between the reference cell and the devices under test

☒ Yes ☐ No — Relative information is provided in method section.

6. Mask/aperture

Size of the mask/aperture used during testing

☒ Yes ☐ No — Device area was determined by a mask with area of 0.09875 cm2. The area of the mask was certified by National Institute of Metrology (NIM, China).

Variation of the measured short-circuit current density with the mask/aperture area

☒ Yes ☐ No — The variation is within 0.3%

7. Performance certification

Identity of the independent certification laboratory that confirmed the photovoltaic performance

☒ Yes ☐ No — The photovoltaic performance of our devices was confirmed by Photovoltaic and Wind Power Systems Quality Test Center, IEE, Chinese Academy of Sciences (PWQTC).

A copy of any certificate(s)
Provide in Supplementary Information

☒ Yes ☐ No — A copy is provided in Supplementary Information.

8. Statistics

Number of solar cells tested

☒ Yes ☐ No — The average PCE of the OSCs is obtained from 30 independent devices.

Statistical analysis of the device performance

☒ Yes ☐ No — We have given statistical data of device performance.

9. Long-term stability analysis

Type of analysis, bias conditions and environmental conditions
For instance: illumination type, temperature, atmosphere humidity, encapsulation method, preconditioning temperature

☒ Yes ☐ No — The thermal stability and the long-term stability measurements of the devices are provided in the manuscript.
